# The architecture and ppGpp-dependent expression of the primary transcriptome of *Salmonella *Typhimurium during invasion gene expression

**DOI:** 10.1186/1471-2164-13-25

**Published:** 2012-01-17

**Authors:** Vinoy K Ramachandran, Neil Shearer, Jobin J Jacob, Cynthia M Sharma, Arthur Thompson

**Affiliations:** 1 Institute of Food Research, Norwich, UK, University of Würzburg, Josef-Schneider-Str. 2/Bau D15, 97080 Würzburg, Germany; 2 Research Center for Infectious Diseases (ZINF), University of Würzburg, Josef-Schneider-Str. 2/Bau D15, 97080 Würzburg, Germany

## Abstract

**Background:**

Invasion of intestinal epithelial cells by *Salmonella enterica *serovar Typhimurium (*S*. Typhimurium) requires expression of the extracellular virulence gene expression programme (ST^EX^), activation of which is dependent on the signalling molecule guanosine tetraphosphate (ppGpp). Recently, next-generation transcriptomics (RNA-seq) has revealed the unexpected complexity of bacterial transcriptomes and in this report we use differential RNA sequencing (dRNA-seq) to define the high-resolution transcriptomic architecture of wild-type *S*. Typhimurium and a ppGpp null strain under growth conditions which model ST^EX^. In doing so we show that ppGpp plays a much wider role in regulating the *S*. Typhimurium ST^EX ^primary transcriptome than previously recognised.

**Results:**

Here we report the precise mapping of transcriptional start sites (TSSs) for 78% of the *S*. Typhimurium open reading frames (ORFs). The TSS mapping enabled a genome-wide promoter analysis resulting in the prediction of 169 alternative sigma factor binding sites, and the prediction of the structure of 625 operons. We also report the discovery of 55 new candidate small RNAs (sRNAs) and 302 candidate antisense RNAs (asRNAs). We discovered 32 ppGpp-dependent alternative TSSs and determined the extent and level of ppGpp-dependent coding and non-coding transcription. We found that 34% and 20% of coding and non-coding RNA transcription respectively was ppGpp-dependent under these growth conditions, adding a further dimension to the role of this remarkable small regulatory molecule in enabling rapid adaptation to the infective environment.

**Conclusions:**

The transcriptional architecture of *S*. Typhimurium and finer definition of the key role ppGpp plays in regulating *Salmonella *coding and non-coding transcription should promote the understanding of gene regulation in this important food borne pathogen and act as a resource for future research.

## Background

Pathogenic strains of *Salmonella *continue to pose an unacceptable worldwide threat to the health of humans and livestock. Infection of humans with *S*. Typhimurium results in a debilitating case of severe gastroenteritis that may result in death in immunocompromised individuals. There are about 1.3 billion cases of non-typhoidal salmonellosis worldwide each year and it is estimated that there are 17 million cases and over 500,000 deaths each year caused by typhoid fever [1]. In the current study we focus on *S*. Typhimurium, which once ingested via contaminated food or water, invades human gut epithelial cells resulting in bloody diarrhoea. *S*. Typhimurium is able to invade intestinal epithelial cells due to the expression of a horizontally acquired set of virulence genes (*Salmonella *Pathogenicity Island 1; SPI1), which encode a type 3 secretion system (T3SS) [2]. In the case of murine infection, *S*. Typhimurium can become systemic and cause a typhoid-like fever due to its ability to replicate and survive within macrophages; this is achieved by the expression of a second T3SS encoded by genes within SPI2 [3]. The complex expression patterns of SPI1 and SPI2 during infection led us and others to develop the concept of the *Salmonella *extracellular (ST^EX^) and intracellular (ST^IN^) virulence gene expression programmes, [4,5] Successful host invasion and colonisation requires expression of the ST^EX ^virulence gene programme followed by expression of the ST^IN ^programme (characterised by SPI1 and SPI2 expression respectively) [6].

The environmentally-dependent expression of nearly all of the ST^EX ^and ST^IN ^genes in *S*. Typhimurium is mediated by the bacterial alarmone, guanosine tetraphosphate (ppGpp) [5]. In *Salmonella *and all beta- and gammaproteobacteria, ppGpp is produced by the activity of two enzymes, RelA and SpoT [for review see [7]]. Whilst RelA is only able to synthesise ppGpp, SpoT contains both synthetase and hydrolase activities. In most other bacteria RelA and SpoT are combined into a single enzyme referred to as Rel or RSH (RelA SpoT homologue) [8]. Previous work implicates SpoT rather than RelA in *Salmonella *pathogenicity since an *S*. Typhimurium Δ*relA *strain is almost fully virulent in BALB/c mouse infection studies, whereas a Δ*relA*Δ*spoT *strain is severely attenuated [9]. It has also been shown that ppGpp plays a key role in coupling virulence to metabolic status in several other pathogenic bacteria including *Mycobacterium tuberculosis *[10,11], *Listeria monocytogenes *[12], *Legionella pneumophilia *[13,14], *Vibrio cholera *[15] and *Pseudomonas aeruginosa *[16]. A complete understanding of the pathways and mechanisms by which ppGpp mediates bacterial virulence may suggest targets for antimicrobial therapies [17].

Guanosine tetraphosphate appears to exert most of its physiological effects by direct or indirect transcriptional control of target genes and binds near the active centre of RNA polymerase (RNAP) to modulate its activity, resulting in the direct repression of stable RNA operons (for review see [18]. This is suggested to increase the availability of RNAP for activation of genes required for survival under various stressful conditions [7]. One mechanism by which this occurs is via sigma factor competition, whereby ppGpp reduces the affinity of core RNAP for σ^70 ^resulting in an increase in the availability of RNAP to bind alternative stress-response sigma factors [19]. Although this model suggests an indirect mechanism for ppGpp activation of gene expression, direct activation has been observed at some promoters [20,21]. The effect of ppGpp on transcription can also be potentiated by the RNAP accessory protein DksA which may help to stabilise the binding of ppGpp to RNAP [22].

Recently global transcriptome analysis using high-density tiling arrays and high throughput RNA sequencing (RNA-seq) has revealed an unexpected complexity of bacterial and archaeal transcriptomes [23-26]. A major advance in this area has been the development of differential RNA sequencing (dRNA-seq) which allows global and unambiguous mapping of transcription start sites (TSSs) [24,27]. In this study we utilise dRNA-seq technology to define the primary transcriptomes of wild-type *S*. Typhimurium and an isogenic Δ*relA*Δ*spoT *mutant, in order to define the extent of ppGpp-dependent expression. We identified primary TSSs for 78% of the annotated *S*. Typhimurium genes as well as ppGpp-dependent and independent alternative TSSs. We confirm the expression of known and predicted sRNAs [28], identify new candidate sRNAs, and report the discovery of 302 candidate antisense transcripts for the entire *S*. Typhimurium genome. Our data provides further insights into the regulatory roles of ppGpp, confirming and extending a previously reported link to global regulation of non-coding RNAs [29]. The high resolution transcriptomic datasets presented here should facilitate future research on transcriptional and post-transcriptional regulation of virulence and other adaptive mechanisms within *Salmonella*.

## Results

### Identification of transcriptional start sites

The nucleotide position of TSSs were identified from a dRNA-seq analysis of RNA samples isolated from the *S*. Typhimurium wild type strain (SL1344) and an isogenic Δ*relA*Δ*spoT *strain grown to early stationary phase (SPI1 inducing conditions). The dRNA-seq analysis was performed according to Sharma *et al *[24]. For each strain two cDNA libraries were prepared from the same total RNA sample. One library, referred to as (+), was enriched for primary transcripts by treating with terminator exonuclease (see Materials and Methods) and the second library, referred to as (-) or non-enriched, was untreated and contained both primary and processed transcripts. Following sequencing of the cDNA libraries on Roche-454 and Illumina-Solexa platforms the reads were mapped onto the SL1344 genome (including the endogenous SLP1-3 plasmids) and the number of reads mapping to each nucleotide position were visualised using the integrated genome browser (IGB; http://bioviz.org/igb/). Elevated read numbers at the 5' end of transcripts in the (+) library relative to the (-) library were identified as an increased presence of transcripts with 5' PPP end sequences compared to 5' P end sequences as described previously [24]. Although 454 sequencing provided longer read lengths, the read numbers of the Solexa dataset were considerably higher and were used primarily for identification of TSSs (see additional file 1: Table S1 for sequencing and mapping statistics).

**Figure 1:**
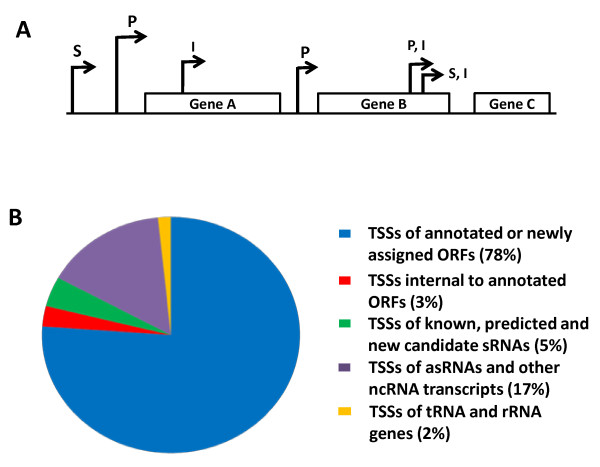
**Annotation of TSSs**. (A) TSSs were defined as primary (P), secondary (S) or internal (I). Primary TSSs were identified by higher read counts relative to secondary TSSs. Internal TSSs were located within the coding region (CDR) of a gene where a TSS was annotated for the gene immediately upstream. Primary and secondary internal TSSs (P,I and S,I respectively) were located within the CDR of a gene where there were no TSSs annotated for the gene immediately downstream. (B) Pie chart showing percentage distribution of TSSs within the *S*. Typhimurium transcriptome.

**Figure 2:**
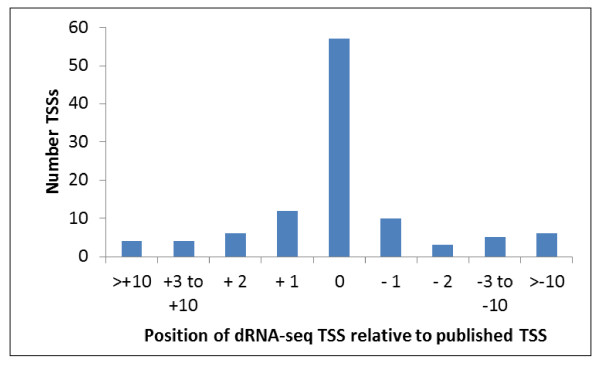
**Comparison of published, experimentally-mapped *S*. Typhimurium TSSs and dRNA-seq mapped TSSs**. The positions of 85% and 92% of the dRNA-seq identified TSSs were located within 2 nt and 10 nt of the experimentally determined TSSs respectively.

We identified a total of 3306 TSS's mapping on to the *S*. Typhimurium SL1344 chromosome (including all ORFs, stable RNAs and ncRNAs) and a further 100 for the SLP1-3 plasmids. TSSs were categorised as primary, secondary, internal or alternative. A definition and summary of the TSS categories for the *S*. Typhimurium genome is shown in Figure 1AB. As reported for *H. Pylori*, many of the different categories of TSSs had multiple associations and this data is summarised in Table 1 (compiled from additional file 2: Tables S1, S2, S3, S4, S5 and S6)[24]. Of the total TSSs, 2398 and 54 were located upstream of all annotated SL1344 ORFs and stable RNAs respectively (Table 1). Primary TSSs were identified for a total of 3581 protein coding genes in 2163 operons (1538 mono and 625 polycistronic) representing 78% of the annotated SL1344 genome (Genebank ID FQ312003.1). It is most likely that the remaining 22% of genes for which no TSS's were mapped were either not expressed under the invasion growth conditions modelled in this study or the mRNA was subject to *in vivo *cleavage by RNAses (e.g. RNaseE). In the latter case the remainder of the transcripts may be ribosome protected resulting in an under-identification of TSSs. In order to validate the identification of TSSs by dRNA-seq, several approaches were utilised. Analysis of the TSSs located directly upstream of annotated ORFs revealed that 75% of the transcripts started with a purine residue (A - 48.5%, G - 25.71%) in accordance with the known preference for a purine residue at the +1 position [30]. Comparison of the dRNA-seq identified TSSs with 107 published *S*. Typhimurium TSSs showed that 90% of the dRNA-seq defined TSSs were within ± 5 nts of the experimentally defined TSSs (Figure 2, additional file 1: Table S3). Lack of concordance between the remaining dRNAseq and experimentally determined TSSs may reflect growth condition related alternative start sites or that experimental techniques do not always distinguish between processed and unprocessed mRNAs. We also used 5' RACE to verify 3 TSSs and to clarify 3 ambiguous TSSs revealing that in each case, the experimentally determined TSSs matched those predicted by dRNA-seq (additional file 3: Figure S1).

**Table 1:** Summary of TSS types and categorisations.

TSS type	ORFs	tRNAs	rRNAs	Known & predicted sRNAs	New candidate sRNAs	Candidate asRNAs	Other candidate ncRNAs
P	1940	38	7	83	47	68	152
S	428	2	7	16	5	1	1
I	95	0	0	0	0	1	1
P,I	114	0	0	0	5	208	32
S,I	14	0	0	0	3	5	0
P,Alt	28	0	0	0	0	0	0
S,Alt	2	0	0	0	0	0	0
P,Alt,I	1	0	0	1	0	0	0
S,Alt,I	1	0	0	0	0	0	0
**Total**	**2623**	**40**	**14**	**100**	**60**	**283**	**186**

Compiled from additional file 2: Tables S1, S2, S3, S4 and S5. P, S, I and Alt refer to primary, secondary, internal and ppGpp-dependent TSSs respectively. Data for SLP1-3 plasmids not shown.

The direct visualisation of transcribed genomic loci and unambiguous mapping of primary TSSs enabled optimisation of the SL1344 genome annotation. Where transcription was observed in regions where no gene was previously annotated the Artemis genome browser and annotation tool (http://www.sanger.ac.uk/resources/software/artemis/) was used to search for potential ORFs possessing upstream Shine-Dalgarno sequences and putative homologues were identified using BLAST (http://blast.ncbi.nlm.nih.gov/Blast.cgi). We applied a similar procedure to re-annotate start codons where the TSS was found to be downstream of the previously annotated start. These procedures resulted in the re-annotation of 60 start codons (additional file 2: Table S9), the identification of 23 potential new ORFs (additional file 2: Table S7), and the re-designation of 2 ORFs previously annotated in different reading frames. Five of the new ORFs (*ibs123 *and *ldrAB*) were predicted to be small toxic peptides of the Type 1 toxin-antitoxin systems found in *E. coli *[31,32].

### Promoter analysis of transcriptional start sites

**Figure 3:**
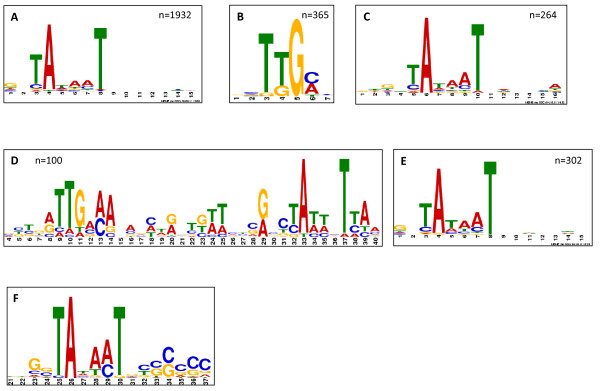
**Conserved motifs identified from promoter regions**. A MEME analysis of 2695 promoters from chromosomal and SLP1-3 SL1344 genes. (A) Conserved -10 motif in 1932 promoters. (B) Conserved -35 motif in 365 promoters. (C) Conserved motif (designated motif 1) in 264 promoters. A MEME analysis of ncRNA promoters revealed (D) conserved -10 and -35 motifs in all of the known and predicted sRNAs (100) and (E) a conserved -10 motif in 280 sites from 302 asRNA promoters. (F) Discriminator region identified from a MEME analysis of 52 ppGpp-repressed promoters (greater than 4-fold).

The dRNA-seq identification of TSSs for the majority of the SL1344 genome enabled us to undertake a MEME based analysis of the promoter regions to identify conserved sequences that may represent binding sites for transcriptional regulatory proteins (e.g. sigma factors). In order to analyse promoter regions, 15 nt sequences upstream of, and including the TSSs, were extracted for all of the 2695 TSSs identified upstream of SL1344 chromosomal and SLP1-3 ORFs (additional file 2: Table S1). The database of promoters was analysed using MEME to identify conserved motifs. From this analysis a conserved σ^70 ^(-10) binding site (TANaaT) was identified for 1932 promoters (Figure 3A). This consensus sequence closely matches the *E. coli *consensus σ^70 ^(-10) binding site (TATAAT) except for decreased conservation at the -11, -10 and -9 positions. A functional category analysis revealed that the highest percentage of promoters that contained conserved -10 regions were upstream of genes encoding vitamins and cofactors (92%), and the lowest percentage encoded motility and chemotaxis related genes (42%; see additional file 3: Figure S2). We found that just over half of the pathogenesis-related genes (57%) contained a conserved -10 region including the major regulators of SPI1 and SPI2, *hilA*, *hilD*, *ssrA *and *ssrB*. By searching 50 nt sequences upstream of the 1932 promoters we were also able to identify a conserved -35 region (TTGaca) for 365 promoters (Figure 3B). A functional analysis of the promoters containing a conserved -35 motif (365 promoters) revealed that by far the highest category (41%) belonged to genes related to cell division (additional file 3: Figure S3).

The dRNA-seq derived promoter database was also exploited to perform a MEME analysis of all of the ORF promoters to identify candidate and known targets for the alternative sigma factors σ^24^, σ^28^, σ^32^, σ^38 ^& σ^54^. Firstly, a position specific probability matrix (PSPM) was derived by MEME analysis for each of *E. coli *alternative sigma factors from a promoter dataset from regulonDB http://regulondb.ccg.unam.mx/. The PSPM was then interrogated with FIMO (Find Individual Motif Occurrences) with a default p-value cut-off of 0.00001 to identify sigma factor specific promoters from the dRNA-seq promoter database. FIMO identified candidate binding sites for σ^24^, σ^28^, σ^32^, σ^38 ^& σ^54 ^factors at 20, 21, 34, 92 and 2 promoters respectively (additional file 2: Table S1). Our analysis was able to identify the majority of the genes previously shown to be dependent on sigma factors (6, 8, 13 and 9 genes for σ^24^, σ^28^, σ^32 ^and σ^38 ^respectively from *S*. Typhimurium or *E. coli*)[33-36]. The published genes shown to be dependent on alternative sigma factors are indicated in additional file 2: Table S1. A MEME analysis was performed on the remaining 592 promoters that did not contain identifiable potential sigma factor motifs. The analysis identified a conserved region for 264 promoters which was similar to the -10 motif, but contained a longer (5 nt) region between the first conserved T of the -10 motif (at -6) and the first nucleotide of the TSS (Figure 3C). A functional categorisation of the 264 promoters revealed that a strikingly high percentage (35%) belonged to genes involved in nucleoside and nucleotide interconversions (see additional file 3: Figure S4).

### 5' leader regions and leaderless mRNAs

Canonical bacterial mRNAs contain a 5' untranslated region (5' UTR) upstream of the initiation codon. As a minimal requirement, this region contains the Shine-Dalgarno (SD) ribosome binding site (RBS), and may contain additional sequence motifs required for efficient ribosome binding [37,38]. In addition, many mRNAs include longer 5' leader regions which may possess regulatory functions. The structure and sequence of 5' leader regions can affect gene expression by modulating the synthesis of full length mRNAs or via regulation of post-transcriptional processing (e.g. synthesis of leader peptides, formation of secondary structures including riboswitches, or binding of proteins or regulatory sRNAs) [39]. In addition to the majority of mRNAs, a few bacterial transcripts lack upstream UTRs and are termed "leaderless transcripts". In such cases transcription starts at, or up to 6 nucleotides upstream of the "A" residue of an AUG start codon [40,41] and the resulting transcripts lack a SD RBS. Published examples include the *cI *repressor of bacteriophage λ [42], and the *tetR *gene of transposon Tn*1721 *[43]. Both the *cl *and *tetR *genes encode relatively low abundance regulatory proteins which is consistent with the reduced translation of genes lacking ribosomal binding sites [44]. Interestingly Sullivan *et al *[45] recently showed that the leaderless mRNA transcript of a regulatory gene, *acuR*, (involved in regulation of an operon encoding products involved in dimethylsulfoniopropionate catabolism in *Rhodobacter sphaeroides*), is transcribed at least as efficiently as downstream genes, but is translated at far lower levels, thus providing an elegant mechanism for differential control of operon-encoded protein levels. Although previously thought to be rare, leaderless genes are now known to be fairly common in prokaryotes and in the archaea, where as many as 69% of protein coding transcripts are leaderless [24,38,46]. Our dRNA-seq analysis identified 16 completely leaderless mRNAs where transcription started precisely at the A residue of the AUG start codon. A further 17 genes contained a leader of between 1 and 6 nt in length but lacked a SD sequence (additional file 2: Table S2). Functional analysis of these 33 leaderless genes showed that 6 encoded transcriptional regulators (including a TetR homologue), expected to be expressed at relatively low levels, 7 encoded membrane proteins which are also often required at low levels, and 4 were related to pathogenicity functions (additional file 2: Table S2).

**Figure 4:**
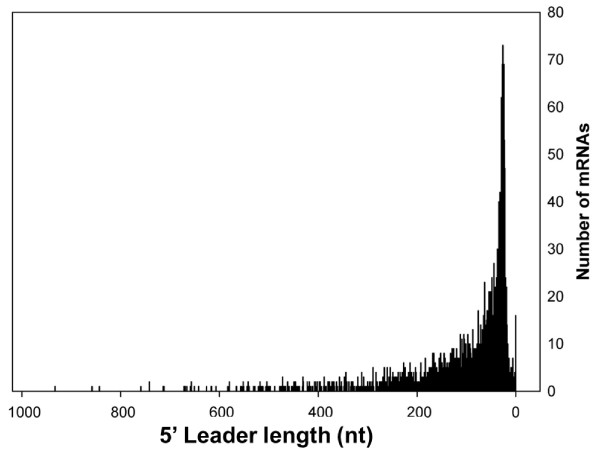
**Length distribution of 5' leader sequences**. The frequency of individual 5' Leader lengths was based on an analysis of 1942 primary and secondary TSSs (additional file 2: Table S1).

**Figure 5:**
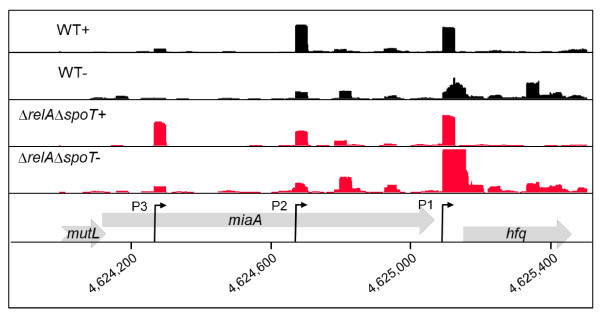
**Promoter architecture of the *S*. Typhimurium *hfq *gene reveals a ppGpp-dependent TSS**. Enriched (+) and non-enriched (-) cDNAs of *S*. Typhimurium wild-type (black) or *ΔrelAΔspoT *(red) strains mapped onto the *miaA*-*hfq *locus. The TSSs are marked by black arrows (P1 - 4625091, P2 - 4624672, P3 - 4624269). The Y axis in each lane represents 0-150 mapped reads per genome position. The genome co-ordinates are shown across the bottom. Note that *hfq *is operonic with the downstream genes, *hflXKC*.

TSS mapping of *S*. Typhimurium wild-type and Δ*relA*Δ*spoT *strains revealed considerable variation in the length of mRNA 5' leader regions ranging from 0-933 nt with a peak at 26 nt and median of 58 nt (Figure 4). We discovered that 735 genes were synthesised from one or more mRNAs containing 5' leaders > 100 nt in length, suggesting that regulatory mechanisms associated with long 5' leaders are widely used by *S*. Typhimurium. As has been reported for *E. coli *[47], no global link between the length of the 5' leader and the functional category of the encoded protein was observed (results not shown). However, some of the longest *S*. Typhimurium 5' leaders were associated with genes involved in global and virulence gene regulation, including *hfq *(887 nt), *lrhA *(712 nt), *invF *(642 nt), *rpoS *(566 nt) and *hilD *(551 nt). One of the longest 5' leaders (887 nt) was transcribed from one of the three promoters regulating the expression of the RNA chaperone *hfq *(Figure 5). The *E. coli hfq *gene is also transcribed from 3 promoters and the TSSs identified by primer extension exactly match our dRNA-seq predicted TSSs in *S*. Typhimurium [48]. The distal *hfq *promoter directing the longest 5' leader is σ^32 ^dependent in *E. coli *and a clear σ^32 ^consensus sequence was found in the corresponding *S*. Typhimurium promoter. Although the role of the leader in regulating gene expression has not yet been defined we found that this promoter was repressed by ppGpp (Figure 5).

### Operon Prediction

Operons were identified during manual inspection of the strand-specific dRNA-seq data (additional file 2: Table S1). We found no difference in the operon structures determined from the wild-type and Δ*relA*Δ*spoT *genomes (data not shown). The majority of 5' ends of operons were assigned from the primary TSS at the start of the first gene or from TSS's located within the first gene of the operon (where present). A further criterion for identification was the presence of 3' UTRs located at the ends of operons. Our inspection identified 1538 monocistronic transcripts and 625 polycistronic operons resulting in a mean of 1.65 genes per operon. We compared our operon map to operons predicted using DOOR (Database of prOkaryotic OpeRons; [49,50]; see additional file 2: Table S1). DOOR predicts operons based on a comparison of 675 prokaryotic organisms and accuracy can reach 90.2% and 93.7% for the *B. subtilis *and *E. coli *genomes respectively (http://csbl1.bmb.uga.edu/OperonDB/DOOR.php). DOOR analysis of the *S*. Typhimurium SL1344 genome predicted 955 operons. Our comparison of dRNA-seq to DOOR predicted operons identified 60% (372) with an exact match and 24 (4%) new operons, not predicted by DOOR. We found 36% (229) of the operons identified from our dRNA-seq data were either extended or shortened (by one or two genes) compared to the DOOR predicted operons. In these cases we found that the dRNA-seq data identified TSSs that were located within operons predicted by DOOR (e.g. *hypA *and *hypD *contain internal TSSs within the *hyp *operon which encodes hydrogenase maturation factors; additional file 2: Table S1 [51]). Since the DOOR algorithm does not take into consideration TSS information, we suggest that our dRNA-seq identified operons are likely to be more accurate than the DOOR predicted operon structures.

### dRNA-seq identification of sRNA expression in *S*. Typhimurium

Manual inspection of the dRNA-seq transcriptome of the wild-type and Δ*relA*Δ*spoT *strains identified a total of 83 predicted and known sRNAs and we discovered a further 55 new candidate sRNAs (Table 1). We validated expression of 3 known (RprA, InvR, GcvB), 1 predicted (STnc1020) and 6 new candidate sRNAs (SLnc0011, SLnc0027, SLP1_ncRNA3, SLP1_ncRNA6, SLP2_ncRNA12, SLP2_ncRNA1) using Northern blotting (see additional file 2: Figure S5, additional file 1: Tables S3 and S4), and verified the presence of conserved -10 and -35 regions for the known and predicted sRNAs (Figure 3D)[28]. In order to further validate the *S*. Typhimurium new candidate and predicted sRNAs we determined whether they were conserved within the recently published *S*. Typhi ncRNA transcriptome [52]. Of the 25 newly identified *S*. Typhi sRNAs, 20 sRNA homologues were found within the SL1344 genome. Of the 20 homologues, 2 new candidate sRNAs, 1 predicted sRNA and 1 asRNA (see following section) were expressed in SL1344 under the growth conditions used in this study (SLnc1039, SLnc1005, STnc560 and SLaRNA0247 respectively; additional file 2: Tables S3, S4 and S5). Interestingly 19% of the known, predicted and new candidate sRNAs have secondary TSSs, indicating that they may be subject to differential regulation (Table 1). Finally, we predicted intrinsic transcriptional terminators for 32 new candidate sRNAs and using TargetRNA software (http://snowwhite.wellesley.edu/targetRNA/) we were also able to predict potential targets (additional file 2: Table S4) [53].

### Extensive antisense transcription in *S*. Typhimurium

Antisense RNAs (asRNAs) have been shown to be particularly abundant in eukaryotes, and recently a large proportion of the primary TSSs have been shown to be antisense to ORFs in *E. coli *and *H. pylori*, suggesting that asRNAs have a widespread regulatory function in bacteria [24,25,54,55]. The dRNA-seq analysis detected 302 potential asRNAs in *S*. Typhimurium which were located directly opposite to coding regions of chromosomal genes (Table 1, additional file 2: Table S5). We also annotated ncRNAs which were within or close to the 3' and 5' UTRs of genes but which could not unambiguously be identified as asRNAs according to our strict definition (Table 1, additional file 2: Table S5). Finally, we found 94 ncRNAs which were located in intergenic regions (i.e. greater than 250 nt from the 3' or 5' ends of a gene) where one or both of the flanking genes were located on the opposing or same strand of the ncRNA (additional file 2: Table S5). For validating the presence of selected candidate antisense and ncRNAs in RNA samples two methods were used, Northern blotting and the more sensitive adapter assisted PCR. We verified the presence of 2 candidate asRNAs, SLasRNA0330 and SLasRNA0183 (additional file 3: Figs. S5 and S6). SLasRNA0330 is opposite to the *sipA *ORF, which encodes a SPI1 effector protein and SLasRNA0183 is opposite to the *ycfQ *ORF which encodes a putative transcriptional repressor (additional file 2: Table S1). The presence of 5 candidate ncRNAs were also verified (the short read lengths precluded classification as asRNAs or sRNAs). The 5 candidate ncRNAs were chosen to be representative of the various locations of ncRNAs on the genome with respect to adjacent genes and included putative transcripts found opposite to either the 5' or 3' ends of genes or classified as opposite intergenic (see additional file 3: Figs S5 and S6). The detection of antisense and ncRNAs using these techniques suggests that the observed antisense transcriptional initiation from the dRNA-seq data is not an artefact of library construction. A functional analysis of the genes opposite to asRNAs revealed that the most highly represented categories contained genes related to pathogenic or chemotaxis and motility functions (additional file 3: Figure S7). Similar to the TSSs located directly upstream of protein coding ORFs, we found that 79% of the candidate asRNA transcripts started with a purine residue (A - 51.40%, G - 27.70%). This provides further evidence that the asRNAs are primary transcripts rather than processing fragments or artefacts of the sequencing protocol, and reflects a recent study in *E. coli *where it was shown that 74% of the asRNA transcripts began with a purine [54].

A MEME analysis of the 302 asRNA TSSs revealed a strongly conserved -10 binding site in 280 promoters (TATAAT), however the -35 site was only weakly conserved (Figure 3E). Since the -35 region has been shown to enhance stability of the RNAP-promoter complex, this could suggest that the majority of asRNAs in *S*. Typhimurium are a consequence of promiscuous transcription initiation, as has been suggested for *E. coli *and eukaryotes [54]. One of the mechanisms by which asRNAs opposing 5'UTRs may inhibit translation is by obscuring the ribosome binding site (RBS) [55]. None of the ncRNAs we identified which were opposite to 5'UTRs appeared to obscure the RBS, however, the short Illumina read lengths may preclude this possibility. Alternately, the ncRNA may prevent transcription via transcriptional interference or attenuation [55]. Indeed, some of the genes that were opposite to asRNAs were transcriptionally silent, suggesting a possible role for asRNAs in their regulation (e.g.SLaRNA310 which is antisense to the 3' end of SL1344_2729). Interestingly we found candidate asRNAs and ncRNAs to 41% of the rRNA genes (see additional file 2: Table S5); similarly in *H. pylori*, ~28% of the tRNA and rRNA genes were found to have antisense TSSs [24]. In addition to stable RNA genes, we discovered putative candidate asRNAs to 18 virulence genes, 9 and 7 of which are located on the opposite strand to SPI1 and SPI2 encoded genes (see additional file 2: Table S5). Their potential role in regulating the expression of these virulence genes is currently being investigated.

### Defining ppGpp-dependent gene expression using dRNA-seq

**Figure 6:**
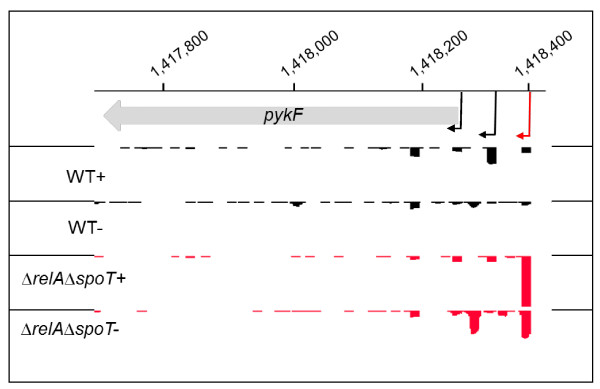
**The *S*. Typhimurium *pykF *gene is expressed from alternative ppGpp-dependent and independent promoters**. Enriched (+) and non-enriched (-) cDNAs of *S*. Typhimurium wild-type (black) or *ΔrelAΔspoT *(red) strains mapped onto the *pykF *gene (encoding pyruvate kinase). The Y axis in each lane represents 0-80 mapped reads per genome position. The genome co-ordinates are shown across the top. Enriched reads show the presence of alternative TSSs in the wild-type strain (black arrow) and the *ΔrelAΔspoT *strain (red arrow). A possible third TSS showing equal read numbers in both wild-type and *ΔrelAΔspoT *strains is indicated by a shorter black arrow.

The dRNA-seq analysis of ppGpp-dependent gene expression identified 32 ppGpp-dependent alternative TSSs (designated 'Alt' in Table 1, additional file 2: Table S1). A functional analysis of the ppGpp-dependent alternative TSSs revealed that 4 were upstream of genes involved in DNA degradation or repair. However, the majority of the genes that were of known function (12 genes) were found to be involved in metabolic processes, e.g., *pykF *which encodes pyruvate kinase is a key glycolytic enzyme, and also able to act as phosphor-donor for nucleoside diphosphates under anaerobic conditions [[56]; Figure 6, additional file 2: Table S1).

**Figure 7:**
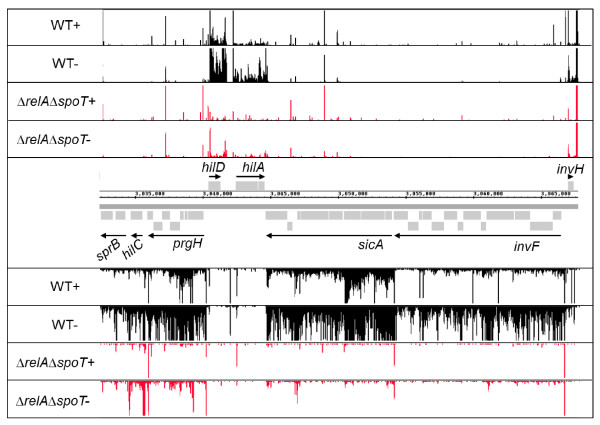
**Promoter architecture and ppGpp-dependency of the *S*. Typhimurium SPI1 pathogenicity island**. Enriched (+) and non-enriched (-) cDNAs of *S*. Typhimurium wild-type (black) or *ΔrelAΔspoT *(red) strains mapped onto SPI1. The Y axis in each lane represents 0-50 mapped reads per genome position. Grey boxes represent individual SPI1 genes and operonic transcripts are indicated by black arrows and labelled according to the first gene of the operon. The relevant genome co-ordinates span the centre of the figure.

Although conventional RNA-seq techniques have been reported to show highly variable coverage across genes and operons, technical modifications have allowed quantitative gene expression studies to be successfully undertaken [52,57,58]. For example, Perkins *et al *[52] used a strand-specific RNA-seq analysis to define the OmpR regulon and validated their results by comparison with conventional microarray experiments. Indeed, our dRNA-seq data shows that it was possible to observe clear differences in SPI1 gene expression between the wild-type and *ΔrelAΔspoT *strains (Figure 7). The expression level of a promoter was determined by calculating the number of non-enriched reads mapped between the primary TSS and 50 nt downstream of the TSS; ppGpp-activated expression was defined as 4-fold or higher transcript levels in the wild-type compared to the Δ*relA*Δ*spoT *strain. ppGpp-repressed expression was defined as 4-fold or higher transcript levels in the Δ*relA*Δ*spoT *strain compared to the wild-type strain. (Table 2, additional file 2: Table S1). The dRNA-seq data revealed that of the genes showing differential expression in the Δ*relA*Δ*spoT *strain the majority of SL1344 ORFs were ppGpp-repressed (752 compared to 131 ppGpp-activated TSSs; Table 2), which may support the suggested role of ppGpp as a passive repressor of transcription [59]. It is possible that the number of ppGpp activated genes was overestimated due to protection of transcripts from degradation by the increased numbers of ribosomes found in ppGpp^0 ^strains growing at low growth rates (e.g. during late-log or stationary phase; [60]). However, since we determined gene expression levels by estimating read numbers from the first 50 nt upstream of the TSS, and the median length of the 5'UTR was 58 nt, any potential effects on expression levels due to ribosome protection will be limited. In order to validate our dRNA-seq based determination of ppGpp-dependent gene expression, we compared the ppGpp-repressed and activated gene sets obtained from dRNA-seq to the filtered ppGpp-dependent gene sets obtained from a whole ORF microarray experiment performed under the same growth conditions and using the same strains (additional file 2: Table S8). Of the dRNA-seq derived ppGpp-repressed and activated genes that were present in the filtered microarray data, 75% and 84% of these were also ppGpp-repressed and activated in the microarray dataset (additional file 2: Table S8). The total number of ppGpp-dependent genes was higher in the dRNA-seq data compared to the microarray data, (752 and 501 genes respectively) reflecting the greater dynamic range of differential expression obtained from dRNA-seq, as has previously been observed [61].

**Table 2:** ppGpp-dependent transcriptional start sites within the *S*.Typhimurium genome

TSSs type	% ppGpp-repressed	% ppGpp activated	Total TSSs
ORF P	29	5	1993
ORF P, I	31	4	116
ORF S	26	7	442
ORF S, I	21	0	14
ORF S, I, Alt	33	0	3
ORF I	21	8	98
Known & predicted sRNAs	7	11	100
New candidate sRNAS	12	11	65
asRNAs	11	11	302
Other ncRNAs	11	8	190
tRNAs	0	40	40
rRNAs	100	0	14

Compiled from additional file 2: Tables S1, S3, S4, S5 & S6.

**Figure 8:**
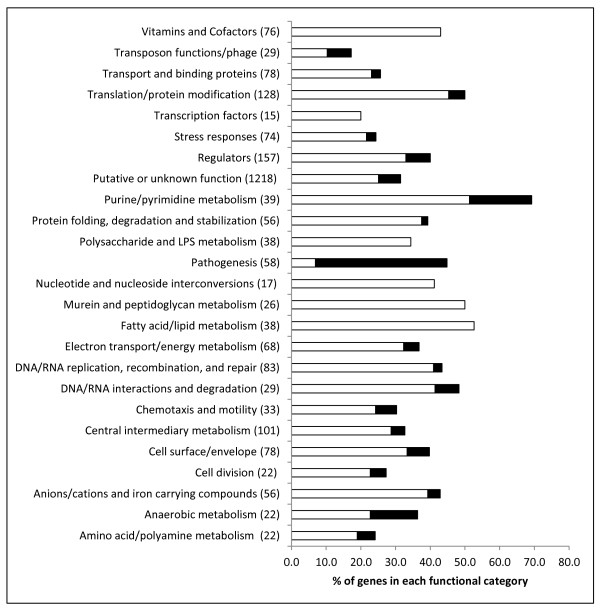
**Functional category analysis of ppGpp-dependent genes**. Functional categories were compiled from the Kyoto Encyclopedia of Genes and Genomes (KEGG; http://www.genome.jp/kegg/) and The Comprehensive Microbial Resource (CMR) at the J. Craig Ventner Institute (http://cmr.jcvi.org/tigr-scripts/CMR/CmrHomePage.cgi) and a manual inspection based on the published literature. Open and shaded bars represent ppGpp-repressed and activated genes respectively. The total number of ORFs present in each category is indicated in parentheses after the category designation.

In order to determine the roles of ppGpp-dependent genes we performed a functional category analysis (Figure 8). We assigned the ppGpp-dependent genes into 25 functional categories. The largest ppGpp-repressed functional categories contained genes related to fatty acid and lipid metabolism, including peptidoglycan metabolism which play a role in the alterations to cell wall structure that occur at the late-log phase of growth. As well as the expected ppGpp-repression of translation related genes, we also observed repression of genes within the categories of pyrimidine and purine metabolism, and DNA/RNA interactions, replication and metabolism. These ppGpp-dependent processes are likely related to adaptation to the decreased growth rate that occurs at late-log phase. We also note that 28 transcriptional regulators were ppGpp-repressed suggesting that some ppGpp-dependent repression may occur via indirect mechanisms. It has been shown that ppGpp-repressed ribosomal RNA genes contain GC-rich discriminator regions located between the TSS and -10 regions that play a role in destabilisation of the RNAP-promoter complex [62,63]. A MEME analysis revealed that 66% of the genes that were ppGpp-repressed by greater than 16-fold contained a conserved 6 nt long GC rich discriminator regions and a Weblogo analysis (http://weblogo.berkeley.edu/) showed a tendency towards C rather than G residues in all 6 positions (Figure 3F). The remaining 34% of the highly ppGpp-repressed genes did not contain GC rich discriminator regions and may therefore be indirectly regulated.

Of the ppGpp-activated genes, by far the largest functional category contained pathogenicity-related genes (22 genes), which supports our previous microarray based analysis of ppGpp-dependent virulence gene regulation in *S*. Typhimurium (Figure 8)[5]. We also found 8 ppGpp-activated genes to possess regulatory functions. These include *rtsA *and *flhD *which encode major transcriptional activators of SPI1 and flagella biosynthesis respectively [64,65]. Previous work has shown that ppGpp-activated genes such as amino acid biosynthetic genes tend to contain AT-rich discriminator regions which allow optimal binding with the σ-subunit of RNAP [62,66]. In confirmation, a MEME comparison of the ppGpp-activated genes revealed a tendency towards AT-rich discriminator regions (an average of 68% AT content for ppGpp activated promoters compared to 57% for all promoters), however no conserved motifs could be identified using MEME.

### ppGpp-dependent expression of non-coding RNAs

Of the total known and predicted sRNAs we found that 18% of the TSSs (18) were ppGpp-dependent out of a total of 100 start sites, and 25% (15) of the new candidate sRNAs were ppGpp-dependent out of a total of 65 start sites (Table 2, additional file 2: Tables S3 and S4). This is less than the proportion of ppGpp-dependent TSSs identified for SL1344 chromosomal ORFs (34%). Similar ppGpp dependent control of small non-coding RNA abundance has been observed in other bacteria including *Rhizobium etli *and *Staphylococcus aureus *[29,67]. Of the total number of ppGpp-dependent known, predicted and new candidate sRNAs, 18 were elevated and 15 repressed by ppGpp (Table 2). As noted previously, a characteristic of ppGpp-repressed genes is the presence of a GC rich discriminator region located between the TSS and the -10 region; however, there was no clear correlation between the GC content of the sRNA discriminator region and fold-repression or activation by ppGpp (data not shown). This suggests that the majority of ppGpp repressed sRNAs may be indirectly regulated, or the size of the dataset was too small to identify a conserved motif. We confirmed the dRNA-seq defined ppGpp-activation of of 2 sRNAs (STnc1020 and InvR) by Northern blotting, one of which (InvR) was previously found to be ppGpp-dependently elevated [68] (additional file 3: Figure S5, additional file 2: Table S3). We also confirmed the dRNA-seq defined ppGpp-repression of RprA (additional file 3: Figure S5, additional file 2: Table S3).

Of the 302 asRNAs that were directly opposite ORFs, we note that 32 were ppGpp-dependently elevated and 32 repressed (Table 2, additional file 2: Table S5). This represents a total of 21% of the candidate asRNAs and is similar to the percentage of ppGpp-dependent sRNAs. Interestingly we note that antisense transcripts to the *sipA *and *invH *genes were ppGpp-activated by 4.3 and 17-fold respectively. The remaining 190 start sites assigned to ncRNAs, (which we could not unambiguously identify as asRNAs) showed a similar level of ppGpp-dependency to the antisense and sRNA TSSs (19%).

## Discussion

We have determined the TSSs for 78% of the *S*. Typhimurium ORFs during growth conditions in which model the extracellular virulence gene expression programme (ST^EX^). To date this is the most extensive and accurate map of the TSSs for this bacterium. Our analysis also identified secondary TSSs for many genes and operon structures. Our MEME based promoter analysis of the first genes of operons identified conserved regions in the promoters which were found to closely resemble consensus binding sites for σ^24^, σ^28^, σ^32^, σ^38 ^& σ^54 ^factors; many of the predicted sigma factor-dependent genes had previously been experimentally verified in either *E. coli *or *Salmonella*. We verified the expression of 38 out of 87 predicted sRNAs and 45 out of 62 known sRNAs [28,68](and J. Vogel; pers. comm.) and also extended the repertoire of sRNAs encoded within the *S*. Typhimurium genome by 55. Of the predicted sRNAs we were unable to verify, it is possible that they were not expressed under the growth condition studied here. We also observed that the location of the TSSs of a subset of the predicted sRNAs did not correspond to the predicted start sites; from this we infer the bioinformatic approach used to identify the TSSs may require experimental-based refinements to enhance accuracy. We identified 302 candidate antisense transcripts for the *S*. Typhimurium genome for which we defined a conserved -10 hexamer upstream of the TSS. Although from this study, we cannot rule out the possibility that the expression of asRNAs are a result of promiscuous transcription, other work suggests this is not the case, at least for *H. pylori *[24].

Our dRNA-seq approach to identifying ppGpp-dependent transcription was validated by comparison with a microarray-based determination of the ppGpp-dependent transcriptome performed under identical growth conditions. The GC rich discriminator region located between the TSS and -10 region of ppGpp-repressed genes has been shown to play a role in destabilising the RNAP-ppGpp complex of rRNA promoters [69]. We were able to correlate decreased transcript levels of ppGpp-repressed genes with the abundance of GC residues within the discriminator region [70]. Our data showed no correlation between AT content of the discriminator region and the level of ppGpp-activation. However in agreement with Da Costa *et al *[66], we did find that in general, ppGpp-activated genes contained a higher overall discriminator AT content. Interestingly we note that SPI1 and SPI2 encoded genes contain AT-rich discriminator regions and the only sigma factor known to contribute to SPI1 expression is σ^70^; this suggests the possibility of a direct activation of SPI1 regulatory genes by ppGpp, rather than via sigma factor competition, as has already been suggested [9,71].

Many regulons controlled by alternative sigma factors, including σ^38 ^and σ^32 ^are poorly induced in cells lacking ppGpp [19]. In order to determine whether this was also the case for *S*. Typhimurium, we analysed our alternative sigma factor promoter database for ppGpp-dependency. We found that almost all the genes belonging to the σ^28 ^and σ^32 ^regulons and more than half of the σ^24 ^-dependent genes were ppGpp-repressed. In contrast, the σ^38 ^regulon showed no tendency towards ppGpp-activation or repression (additional file 2: Table S1). Previous work has also shown that, in contrast to *E. coli*, ppGpp does not control RpoS levels in *S*. Typhimurium during late-log and stationary phase growth [9]. We conjecture that the ppGpp-repression of some of the alternative sigma factor regulons may represent an adaptation to favour σ^70 ^dependent virulence gene expression under the ST^EX ^growth conditions studied here.

It is generally accepted that elevated levels of ppGpp during amino acid starvation (stringent response) result in repression of stable RNAs (rRNA and tRNA). Consistent with this we observed repression of the rRNA operons in the wild-type compared to the Δ*relA*Δ*spoT *strain (additional file 2: Table S6). However, all but one of the tRNA mono- and polycistronic operons showed elevated transcript levels in the wild-type compared to the Δ*relA*Δ*spoT *strain; a similar ppGpp-dependent elevation of tRNA levels was found in stationary phase *Rhizobium etli *relative to early exponential phase [29]. It is possible that elevation of tRNA levels could be a consequence of ppGpp-dependent differential processing or stability rather than direct ppGpp-dependent regulation. Indeed tRNA has been reported to remain stable under starvation conditions that induce rRNA degradation in *E. coli *[72]. In support of the possibility of ppGpp-dependent differential processing or stability of tRNAs we observed that expression of RNaseP, a ribozyme responsible for 5' end processing of tRNAs, was ppGpp-activated in *S*. Typhimurium (additional file 2: Table S1). Similarly, we note that the *R. etli *RNaseP was also ppGpp-activated (29). We hypothesise that the ppGpp-dependent activation of RNaseP may result in reduced tRNA processing in the Δ*relA*Δ*spoT *strain and subsequent removal of incorrectly processed tRNAs via RNA quality control mechanisms [73].

For the known and predicted sRNAs described in this study, a MEME analysis was able to identify conserved -10 (TATTNT) and -35 (TTGaCA) regions upstream of the predicted TSSs (Figure 3D). A manual inspection of the smaller new candidate sRNA dataset identified AT rich -10 hexamers in 69% of the promoters (data not shown). A manual inspection of all of the sRNA promoters described in this study failed to find any of the well-defined alternative sigma factor binding motifs and in fact only four sRNAs, (MicA, RybB, GlmZ and GlmY) have so far been shown to be positively controlled by σ^E ^and σ^54 ^in *E. coli *[74]. This suggests that, at least for the sRNAs transcribed under the growth conditions studied here, their expression is mostly σ^70 ^dependent and perhaps reflects the major role sRNAs play in maintaining house-keeping functions and regulating virulence determinants. In contrast to the discriminator regions of ppGpp-repressed genes, we were unable to identify a conserved GC rich region in the set of ppGpp-repressed sRNAs. Several of the ppGpp-repressed sRNAs (OmrA, OmrB, MicA, MicF and CyaR) have been shown to act as repressors of genes encoding porins and outer membrane proteins (OMPs) suggesting that ppGpp may indirectly activate these target genes. OMPs are important virulence factors and play a significant role in the bacterial adaptation to environmental conditions. Other highly ppGpp-repressed sRNAs shown to play a role in *Salmonella *virulence include IsrI, IsrP and CsrB. In addition, the sRNA chaperone, Hfq was ppGpp-repressed by 5.6-fold thus expanding the role of ppGpp in the regulation of *Salmonella *virulence gene expression (Figure 5) [69]. The IsrI and IsrP sRNAs are expressed during infection of J774 macrophages [75]. IsrI is also expressed during stationary phase, and under low oxygen or magnesium levels; IsrP is expressed under low magnesium and extreme acid conditions of pH2.5 [75]. CsrB is part of the *csr *system shown to play a role in the regulation of invasion gene expression in *S*. Typhimurium [76]. The SPI1 encoded sRNA, InvR, has previously been reported to be ppGpp-activated [28]. Our data confirms InvR as the most highly ppGpp-activated sRNA we detected under these growth conditions (12.5-fold; additional file 2: Table S1). Although Hfq has been shown to reduce the stability of InvR [68], we note that despite a 5.6-fold ppGpp-dependent repression of Hfq trasncript levels, InvR remains highly ppGpp-activated. This suggests that ppGpp is able to modulate InvR transcript levels via a Hfq independent mechanism. InvR represses synthesis of the major outer membrane protein, OmpD [28]. It is suggested sRNAs such as InvR have evolved to modulate OMP levels, which can be deleterious to the cell [77]. OmpD has also been shown to facilitate *Salmonella *adherence to human macrophages and intestinal epithelial cell lines [78,79]. Potential targets for the new ppGpp-dependent sRNAs include *fabH*, involved in the initiation of fatty acid biosynthesis, 6 genes involved in transport of sugars, nitrite, peptides and branched chain amino acids, and 3 transcriptional regulators, *nadR*, *rob*, and STM2275 (see additional file 2: Table S4).

We discovered extensive antisense transcription within the *S*. Typhimurium genome under the growth conditions studied here. Similarly, a considerable abundance of asRNA transcription was also discovered in *E. coli *and *H. pylori *[24,54]. Interestingly, we observed candidate asRNAs to several virulence genes from SPI1, 2 and 6 and identified 4 putative ncRNAs which were classified as opposite intergenic between genes encoding several major SPI1 regulators including *hilA *and *hilD *(additional file 2: Table S5). One of the two candidate ncRNAs between *hilA *and hilD (Sla0508) was highly ppGpp-activated by a factor of at least 34-fold. HilD has been implicated in cross-talk between SPI1 and SPI2 expression [80]. Under the growth conditions used in this study, SPI2 is not highly expressed compared to SPI1. It is therefore tantalising to suggest that ncRNAs may play a role in modulating expression of the ST^EX ^and ST^IN ^virulence gene expression programmes.

## Conclusions

Here we used dRNA-seq to define the transcriptomic architecture for an *S*. Typhimurium wild-type and ppGpp^0 ^strain during growth conditions where the invasion (SPI1) genes are expressed. We identify the precise location of the TSSs for 78% of the *S*. Typhimurium genome, the reannotation of 60 start codons and the identification of 23 potential new ORFs. The nucleotide position of the TSSs enabled us to perform a promoter analysis, which resulted in the prediction of binding sites for 6 sigma factors, the analysis of 5' leader lengths and the prediction of 625 operons. The definition of the ncRNA transcriptome resulted in confirmation of the expression of 83 predicted and known sRNAs under these growth conditions and the prediction of 55 new candidate sRNAs, for which potential targets were inferred. Extensive asRNA transcription was also discovered for 302 candidate asRNAs, 18 and 11 of which were opposite virulence genes and candidate sRNAs respectively. The dRNA-seq predicted ppGpp-dependent TSSs and ppGpp-dependent expression for the SL1344 genome and we showed that ppGpp is involved in regulating an average of 20% of *S*. Typhimurium ncRNA expression.

## Methods

### Bacterial Strains and Growth Conditions

The wild-type, virulent *S*. Typhimurium strain SL1344 (31) was provided by F. Norel (Institut Pasteur) and re-isolated from the spleens of infected BALB/c mice. The SL1344 Δ*relA*Δ*spoT *strain was a kind gift from Dr. Karsten Tedin, Freie Universität, Berlin, and was constructed by lambda red mutagenesis [81]. Deletion of the *relA *and *spoT *genes and intactness of flanking regions were determined by sequencing; antibiotic resistance markers were removed after construction (pers. comm. Dr. K. Tedin). Bacterial cultures were grown overnight in Luria-Bertani broth (LB) at 37°C, 250 rpm, from -70°C glycerol stocks and used to inoculate into 50 ml of fresh LB in 250 ml conical flasks. The cultures were grown aerobically at 37°C with shaking at 250 rpm to an OD_600 _of 2.1, (Early Stationary Phase; ESP), conditions previously shown to induce SPI1 gene expression [82]. We confirmed that the wild-type and Δ*relA*Δ*spoT *strains have almost identical growth rates in LB under these growth conditions (additional file 3: Table S8; 9) and that the wild-type is highly invasive compared to the Δ*relA*Δ*spoT *strain in a HeLa cell infection assay (additional file 3: Table S9; 82).

### RNA Extraction and Purification

Bacterial cultures were harvested at ESP, added to one-fifth volume of stop solution (5% (v/v) phenol in ethanol), and incubated on ice for 30 min to stabilize total RNA [5]. Bacterial cells were harvested at 10,000 rpm at 4°C for 5 min and re-suspended in 500 μl of re-suspension buffer (25 mM Tris-HCl pH7.4, 1 mM EDTA). An equal volume of lysis solution (0.6 M Sodium acetate pH5.2, 4 mM EDTA, 3% SDS) was added and the mixture was boiled for 30 - 60 sec or until the suspension cleared. The solution was then incubated at 20 to 25°C for 5 min before extracting twice with phenol and chloroform. Total RNA was precipitated overnight at -20°C with 2.5 volumes of ice-cold ethanol and pelleted at 13,500 rpm for 60 min at 4°C. The pellet was washed once with 70% ethanol and vacuum dried for 5 min. Finally, the pellet was resuspended in 100 μl of nuclease free water. Chromosomal DNA was removed by digestion with 50-100 units of Turbo DNA-free DNase (Ambion). The removal of contaminating DNA was verified by performing PCR using primers targeted to bacterial housekeeping genes. The quantity and quality of the total RNA was determined using a 2100 Bioanalyzer™(Agilent) before and after DNase treatment.

### Library preparation and sequencing

To differentiate primary transcripts with a 5' triphosphate end (5' PPP) from processed transcripts with a 5' monophosphate end (5' P), total RNA from each strain was divided into equal amounts and one half was treated with Terminator™ 5'-phosphate-dependent exonuclease (TEX, Epicentre Biotechnologies) as previously described [24]. TEX specifically degrades RNAs with a 5' P end but does not degrade transcripts with a 5' PPP end and therefore enriches for primary transcripts. Both the untreated (Non-enriched (NE)) and treated (enriched (EN)) samples were then treated with Tobacco acid pyrophosphatase (TAP; Epicentre Biotechnologies) to generate 5'-mono-phosphate ends for linker ligation. After 5' linker ligation and poly(A) tailing, strand-specific cDNA libraries were constructed by Vertis Biotechnology, AG, Germany (http://www.vertis-biotech.com) as described [24]. For each Roche-454 and Illumina-Solexa sequencing run, four strand specific cDNA libraries were prepared: Wild-type non-enriched (Wt-NE), Wild-type enriched (Wt-EN), Δ*relA*Δ*spoT *non-enriched (DM-NE) and Δ*relA*Δ*spoT *enriched (DM-EN). Each library was tagged with a different barcode at the 5' end to enable multiplexing during sequencing. Four of the Roche-454 cDNA libraries were pooled and sequenced (Liverpool University, UK), yielding a total of ~400,000 reads. Similarly, four of the Illumina-Solexa libraries were pooled and sequenced in a single lane using 36 single-read cycles on an Illumina Genome Analyzer II sequencing machine (GATC Biotech, Germany), yielding a total of ~10^7 ^reads.

### Analysis of sequences and statistics

The mapping statistics for both the Roche-454 and Illumina-Solexa cDNA libraries are shown in additional file 1: Table S1. Following sequencing, custom PERL scripts were used to separate the cDNA libraries based on their barcodes and to remove 5' linker regions. Any Roche-454 sequencing reads less than 12 nt in length were removed to avoid mapping errors. Both Roche-454 and Illumina-Solexa generated reads were mapped onto the *S*. Typhimurium SL1344 genome (Genbank ID. FQ312003.1) including the three native virulence plasmids (SLP1, SLP2 and SLP3) using the *segemehl *program [83]. Mapped reads were converted to a graph file and visualized on an Integrated Genome Browser (IGB) [84].

### Northern Blotting

Northern blotting was performed essentially as described in [24]. Hybridization was performed with 5 pmol [γ-32P]-ATP end-labeled oligodeoxynucleotides (additional file 1: Table S4).

### 5' RACE determination of selected TSSs

The transcriptional start sites of selected genes were determined using 5' RACE as described previously [85]. Briefly, 12 μg of total RNA was treated with TAP and the RNA oligonucleotide adaptor A3 was ligated to the 5' end of the treated RNA. TAP cleaves the 5'-triphosphate of primary transcripts to a monophosphate, thus making them available for ligation of the RNA adaptor. This results in an enrichment of 5'-RACE products for primary transcripts in TAP treated RNA, compared to an untreated control. First strand cDNA synthesis was performed using either random hexamers oligonucleotide primers (for SL1344_1204 and SL1344_1122) or gene-specific primers followed by PCR amplification with nested gene-specific primers and 5' Adaptor-specific DNA primer B6. Resulting PCR products were cloned into the pGEM^®^-T Easy vector (Promega) and sequenced using standard protocols. All primers used are detailed in additional file 1: Table S2.

### Adaptor assisted RT-PCR to detect asRNAs

In general we found that Northern blotting failed to detect non-coding RNAs (ncRNAs) which had Solexa read numbers of less than 200. We therefore employed an RT-PCR technique to selectively amplify predicted ncRNAs and thus confirm their existence. Primers complementary to the 3' end of putative ncRNA but including a universal 5' adaptor sequence were used to prime cDNA synthesis from total RNA samples. RNA (0.5 μg) was mixed with 2 pmol of the RT primer and reverse transcribed at 55°C for 1 hour using AffinityScript (Agilent) according to the manufacturer's instructions. Following heat denaturation of the enzyme, 2 μl of the reaction was used as a template in a PCR reaction using a primer matching the 5' end of the putative ncRNA and a second universal primer (U5), matching the 5' adaptor sequence of the RT primer. For each target, PCR reactions were carried out with cDNA from both wild-type and Δ*relA*Δ*spoT *strains as well as a genomic DNA negative control. Primers used are listed in additional file 1: Table S4.

### Microarray analysis

Microarray analysis was performed as described previously [5]. RNA was extracted from *S*. Typhimurium SL1344 wild type and isogenic Δ*relA*Δ*spoT *strains as described above under identical growth conditions and grown to the same OD. The RNA was labelled and hybridised to IFR SALSA2 whole ORF microarrays (http://www.ifr.ac.uk/Safety/Microarrays/default.html#protocols), and data processed and analysed using GeneSpring™ (Agilent). The data was from 3 biological replicates, statistically filtered (P = 0.05) and a 2-fold cut off applied.

### Microarray accession number

The microarray data discussed in this publication are MIAME compliant and have been deposited in NCBI's Gene Expression Omnibus and are accessible through GEO Series accession number GSE34269 (http://www.ncbi.nlm.nih.gov/geo/query/acc.cgi?acc=gse34269).

## Abbreviations

RNA-seq: RNA sequencing - transcriptomic studies utilising high-throughput deep sequencing of cDNA libraries; dRNA-seq: differential RNA sequencing - RNA-seq based technique that differentiates between primary and processed transcripts; ppGpp: guanosine tetraphosphate; TSSs: transcription start site(s); ORFs: open reading frame(s); ncRNA: non-coding RNA; sRNA: small RNA; asRNA: anti-sense RNA; SPI: *Salmonella *pathogenicity island; RNAP: RNA polymerase; SLP1-3: *Salmonella *plasmids 1-3; IGB: integrated genome browser; BLAST: basic local alignment search tool; UTR: untranslated region; SD: Shine Dalgarno sequence; RBS: ribosome binding site; T3SS: type 3 secretion system; ST^EX ^ST^IN^: *Salmonella *extracellular and intracellular virulence gene expression programs respectively; TEX: terminator exonuclease; TAP: tobacco acid pyrophosphatase.

## Competing interests

The authors declare that they have no competing interests.

## Authors' contributions

AT and VKR designed the study. VKR prepared RNA for sequencing and VKR and CSM wrote custom PERL scripts for mapping and data analysis. NS performed experimental validation of dRNA-seq data. AT, VKR, JJJ and NS manually identified TSSs. AT, VKR and NS wrote the manuscript. All authors read and approved the final manuscript.

## Supplementary Material

Additional file 1 **Supplementary Tables (S1, S2, S3, S4)**. Table S1: Mapping statistics for wild-type and Δ*relA*Δ*spoT *libraries. Table S2: Primers used for 5' RACE identification of TSSs. Table S3: Comparison of published *Salmonella *TSSs and dRNA-seq TSSs. Table S4: Probes and primers used for detection of ncRNAs. Click here for file

Additional file 2 **Supplementary Tables S1, S2, S3, S4, S5, S6, S7, S8, S9**. Table S1: Master table of TSSs and ppGpp-dependent expression levels for *S*. Typhimurium SL1344 ORFs. Table S2: Leaderless transcripts. Table S3: TSSs and ppGpp-dependent expression of known and predicted small RNAs. Table S4: TSSs and ppGpp-dependent expression of new candidate small RNAs. Table S5: TSSs and ppGpp-dependent expression of candidate antisense RNAs. Table S6: TSSs and ppGpp-dependent expression rRNAs and tRNAs. Table S7: Predicted new ORFs. Table S8: Comparison of ppGpp-dependent expression from microarray and dRNA-seq data. Table S9: Re-annotated ORFs. Click here for file

Additional file 3 **Supplementary Figures S1, S2, S3, S4, S5, S6, S7, S8, S9**. Figure S1: 5' RACE identification of transcriptional start sites. Figure S2: Functional category analysis of 1932 promoters of annotated SL1344 ORFs that contain a predicted -10 motif. Figure S3: Functional category analysis of 1932 promoters of annotated SL1344 ORFs that contain a predicted -10 and -35 motif. Figure S4: Functional category analysis of 264 promoters of annotated SL1344 ORFs that contain conserved motif 1. Figure S5: Northern Blot detection of non-coding RNAs. Figure S6: Adapter assisted PCR detection of asRNAs. Figure S7: Functional category analysis of ORFs opposite to candidate asRNAs. Figure S8: Growth curves for *S*. Typhimurium SL1344 wild-type Δ*relA*Δ*spoT *strains. Figure S9: Invasion of *S*. Typhimurium SL1344 wild-type and isogenic Δ*relA*Δ*spoT *strains in HeLa cells at 2 h and intracellular replication at 6 h post-infection. Click here for file
