# Supplementary material for: The architecture and ppGpp-dependent expression of the primary transcriptome of Salmonella Typhimurium during invasion gene expression
Source: BMC Genomics. 2012 Jan 17;13:25. doi: 10.1186/1471-2164-13-25 (PMC3293720; doi:10.1186/1471-2164-13-25)
Supplement: Additional file 1 — Supplementary Tables (S1, S2, S3, S4). Table S1: Mapping statistics for wild-type and ΔrelAΔspoT libraries. Table S2: Primers used for 5' RACE identification of TSSs. Table S3: Comparison of published Salmonella TSSs and dRNA-seq TSSs. Table S4: Probes and primers used for detection of ncRNAs. [file 1471-2164-13-25-S1.DOC]

**Table S1. Mapping statistics for *S*. Typhimurium wild-type and ∆*relA*∆*spoT*** libraries.

| **cDNA libraries** | **Total reads** | **% Mapped reads** | **% of Unmapped reads** |
| --- | --- | --- | --- |
| **Roche-454** |  |  |  |
| *S*.Typhimurium Wt – Non-enriched | 93337 | 59.17 | 40.83 |
| *S*.Typhimurium Wt – Enriched | 111462 | 88.86 | 11.14 |
| *S*.Typhimurium ppGpp^0^ – Non-enriched | 127067 | 83.35 | 16.65 |
| *S*.Typhimurium ppGpp^0^ – Enriched | 94074 | 87.67 | 12.33 |
|  |  |  |  |
| **Illumina-Solexa** |  |  |  |
| *S*.Typhimurium Wt – Non-enriched | 2748299 | 60.75 | 39.25 |
| *S*.Typhimurium Wt – Enriched | 3510529 | 86.64 | 13.36 |
| *S*.Typhimurium ppGpp^0^ – Non-enriched | 1331663 | 85.85 | 14.15 |
| *S*.Typhimurium ppGpp^0^ – Enriched | 1998008 | 88.40 | 11.60 |

Wt - wild-type, ppGpp^0^ - ∆*relA*∆*spoT*. See Methods for definitions of non-enriched and enriched libraries.

**Table S2. Primers used for 5’ RACE determination of transcriptional start sites of selected genes.**

| **TSS** | **Primer sequence (5’ – 3’)** |
| --- | --- |
| Primer B6 | GCGCGAATTCCTGTAGA |
| RNA adaptor A3 | AUAUGCGCGAAUUCCUGUAGAACGAACACUAGAAGAAA |
| SL1344_0602_PCR | CCTCATGCTGTAAATGTTCTT |
| SL1344_1122_PCR | CGATGGCCTCGTGACTATT |
| SL1344_1167_PCR | GCTAACGAGTCGGCTGTC |
| SL1344_1204_PCR | CGTGTGAAATCCTCTATCGG |
| SL1344_3569_PCR | GGTATGAATACCCAGCAGCA |
| SL1344_0602_RT | GCGTTTTAATTCGTGAAGGA |
| SL1344_1122_RT | Random primers |
| SL1344_1167_RT | CCAGATGATCGTAGCTTTCG |
| SL1344_1204_RT | Random primers |
| SL1344_3569_RT | CCTGCTCAAGTTGTTCCATG |

**Table S3. Comparison of published (primer extension) *Salmonella* TSSs and dRNA-seq TSSs.**

| **Gene ID** | **Gene Name** | **Published TSS** | **Strain** | **dRNAseq TSS** | **Reference** |
| --- | --- | --- | --- | --- | --- |
| SL1344_0210 | *htrA* | 244441 | SL1344 | 244441 | 25 |
| SL1344_0394 | *brnQ* | 452569,70,72 | SL2 | 452570 | 36 |
| SL1344_0491 | *copA* | 559995 | 14028s | 559995 | 10 |
| SL1344_0545 | *fimW* | 612821 | 1344 | 612821 | 55 |
| SL1344_0596 | *ahpC* | 669913 | LT2 | 669913 | 53 |
| SL1344_0897 | *lrp* | 993021 | SL1344 | 993011 | 32 |
| SL1344_1027 | *pipB P2* | 1133696-7 | SL1344 | 1133696 | 22 |
| SL1344_1027 | *pipB P1* | 1133831 | SL1344 | 1133876 | 22 |
| SL1344_1030 | *sopB (sigD)* | 1136295-6 | SL1344 | 1136295 | 8 |
| SL1344_1100 | *pyrC* | 1205738 | LT2 | 1205738 wt | 50 |
| SL1344_1100 | *pyrC* | 1205735 | LT2 | 1205736 DM | 50 |
| SL1344_1109 | *flgM* | 1213533 | LT2 | 1213533 | 13 |
| SL1344_1110 | *flgA* | 1214256 | KK2040 | 1214257 | 17 |
| SL1344_1111 | *flgB* | 1214366-7 | KK2040 | 1214364 | 17 |
| SL1344_1120 | *flgK* | 1221586 | KK1004 | 1221586 | 23 |
| SL1344_1163-59 | *potABCD* | 1269375 | LT2 | 1268374 | 29 |
| SL1344_1164 | *pepT* | 1269538 | LT2 | 1269538 | 29 |
| SL1344_1164 | *pepT* | 1269451 | LT2 | 1269451 | 29 |
| SL1344_1169 | *phoP* | 1276250 | 14028s | 1276250 DM | 24 |
| SL1344_1169 | *phoP* | 1276239 | 14028s | 1276239 wt | 49 |
| SL1344_1180 | *msgA* | 1286623 | 14028 | 1286653 | 14 |
| SL1344_1180 | *msgA* | 1286623 | 14028 | 1286584 | 14 |
| SL1344_1183 | *pagD* | 1288331 | 14028s | 1288331 | 24 |
| SL1344_1184 | *pagC* | 1288545 | 14028s | 1288534 | 24 |
| SL1344_1187 | *agsA* | 1292311 | M556 | 1292311 | 56 |
| SL1344_1266 | *rfc (wzy)* | 1368311 | C52 | 1368311 | 58 |
| SL1344_1325 | *ssrB* | 1433807 | 14028s | 1433807 | 11 |
| SL1344_1326 | *ssrA* | 1436617 | 14028s | 1436618 | 11 |
| SL1344_1327 | *ssaB (spiC)* | 1436851 | 14028s | 1436851 | 57 |
| SL1344_1331 | *sseA* | 1440403-4 | 14028s | 1440133 | 57 |
| SL1344_1340 | *ssaG* | 1446354 | 14028s | 1446354 | 57 |
| SL1344_1340 | *ssaG* | 1446353 | SF530 UK1 | 1446354 | 26 |
| SL1344_1376 | *slyA* | 1476696 | ST12/75 | 1476693 | 51 |
| SL1344_1377 | *slyB* | 1477787 | 14028s | 1477787 | 24 |
| SL1344_1442 | *dcp* | 1546519 | LT2 | 1546519 | 15 |
| SL1344_1450-48 | *marRAB* | 1554813-4 | LT2 | 1554812 | 52 |
| SL1344_1530 | *pcgL* | 1647623 | 14028s | 1647622 | 24 |
| SL1344_1531 | *ugtL* | 1648673 | 14028s | 1648668 | 48 |
| SL1344_1590 | *ogt* | 1710445-6 | C52 | 1710445 | 16 |
| SL1344_1639 | *pyrF* | 1758231 | LT2 | 1758228 | 54 |
| SL1344_1645 | *cysB* | 1766328 | LT2 | 1766328 | 39 |
| SL1344_1663 | *ompW* | 1785413 | 14028s | 1785413 | 12 |
| SL1344_1705 | *hemA* | 1832601 | LT2 | 1832601 | 5 |
| SL1344_1705 | *hemA* | 1832694-3 | LT2 | 1832692 | 5 |
| SL1344_1705 | *hemA P1* | 1832694 | SL1344 | 1832692 | 9 |
| SL1344_1705 | *hemA P2* | 1832602 | SL1344 | 1832601 | 9 |
| SL1344_1706 | *lolB* | 1832739-8 | LT2 | 1832739 | 5 |
| SL1344_1769 | *mgrB (yobG)* | 1894655 | 14028s | 1894655 | 24 |
| SL1344_1784 | *sopE2* | 1910341 | SL1344 | 1910324 | 28 |
| SL1344_1797 | *pagM* | 1919906 | 14028s | 1919904 | 24 |
| SL1344_1799 | *pagK* | 1920692 | 14028s | 1920693 | 24 |
| SL1344_1849 | *flhB* | 1969024 | KK2040 | 1969025 | 17 |
| SL1344_1860 | *flhD P1* | 1979837 | KK1004 | 1979837 | 62 |
| SL1344_1860 | *flhD P3* | 1979930 | KK1004 | 1979930 | 62 |
| SL1344_1860 | *flhD P5* | 1980164 | KK1004 | 1980165 | 62 |
| SL1344_1860 | *flhD P4* | 1979986 | KK1004 | 1979990 | 62 |
| SL1344_1885 | *fliA* | 2003063 | KK2040 | 2003063 | 18 |
| SL1344_1888 | *fliC* | 2006775-6 | LT2 | 2006774 | 1 |
| SL1344_1889 | *fliD* | 2006916 | KK1004 | 2006916 | 23 |
| SL1344_1897 | *fliE* | 2013913 | KK2040 | 2013915 | 17 |
| SL1344_1904 | *fliL* | 2020599 | KK2040 | 2020598 | 17 |
| SL1344_1924 | *cspB* | 2037150 | SL1344 | 2037150 | 7 |
| SL1344_2011 | *cob operon* | 2111978 | LT2 | 2111978 | 44 |
| SL1344_2011 | *cob operon* | 2111917 | LT2 | 2111918 | 44 |
| SL1344_2057 | *ugd* | 2156962 | 14028s | 2156959 | 35 |
| SL1344_2241 | *gyrA* | 2374100 | SL1344 | 2374100 | 20 |
| SL1344_2265 | *pmrG* | 2401492 | 14028s | 2401492 | 59 |
| SL1344_2266 | *pbgP* | 2401611 | 14028s | 2401611 | 59 |
| SL1344_2273 | *pmrD* | 2409318 | 14028s | 2409317 | 24 |
| SL1344_2297-2285 | *nuoA-N* | 2437519 | LT2 | 2437519 | 2 |
| SL1344_2297-2285 | *nuoA-N* | 2437342 | LT2 | 2437342 | 2 |
| SL1344_2393 | *cysK* | 2540914 | LT2 | 2540915 | 4 |
| SL1344_2413-2414 | *amiI, hemF* | 2557942 | LT2 | 2557941 | 60 |
| SL1344_2604 | *rpoE* | 2780568 | SL1344 | 2780568 | 34 |
| SL1344_2604 | *rpoE* | 2780635 | SL1344 | 2780633 | 34 |
| SL1344_2604 | *rpoE* | 2780492 | SL1344 | 2780494 | 34 |
| SL1344_2674 | *sopE* | 2862878 | SL1344 | 2862878 | 8 |
| SL1344_2790-1 | *nrdEF* | 2974367 | LT2 | 2974367 | 19 |
| SL1344_2794-6 | *proVWX* | 2978274 | LT2 | 2978274 | 43 |
| SL1344_2847 | *hilC* | 3035598 | SL1344 | 3035598 | 37 |
| SL1344_2854 | *prgH* | 3040128 | 1344 | 3040128 | 30 |
| SL1344_2855 | *hilD* | 3040377-8 | SL1344 | 3040377 | 46 |
| SL1344_2856 | *hilA* | 3042082 | LT2 | 3042084 | 45 |
| SL1344_2865 | *sicA* | 3054030 | SL1344 | 3054030 | 8 |
| SL1344_2878 | *invF* | 3066639 | 1344 | 3066639 | 30 |
| SL1344_2903 | *rpoS* | 3089613 | Dublin & LT2 | 3089613 | 41 |
| SL1344_2927-2925 | *cysJIH* | 3114694 | LT2 | 3115592 | 38 |
| SL1344_2962 | *gcvA* | 3156722 | SL1344 | 3156722 | 47 |
| SL1344_3150 | *ygiW* | 3361510 | 14208 | 3361383 | 33 |
| SL1344_3151 | *preAB (QseBC)* | 3361078-9 | 14208 | 3361475 | 33 |
| SL1344_3217 | *tdcA* | 3434146 | SL1344 | 3434145 | 21 |
| SL1344_3357-58 | *yhdG-fis P2* | 3576659 | LT2 | 3576659 | 40 |
| SL1344_3566 | *yhiV* | 3795733 | LT2 | 3795863 | 63 |
| SL1344_3579 | *dctA* | 3812934 | LT2 | 3812934 | 3 |
| SL1344_3729 | *mgtC* | 3986781 | 14028s | 3986780 | 24 |
| SL1344_3759-3757 | *deoKPX* | 4015862 | LT2 | 4015861 | 6 |
| SL1344_3760 | *deoQ* | 4016076 | LT2 | 4016076 | 6 |
| SL1344_3918 | *metR* | 4191099 | JL781 | 4191097 | 42 |
| SL1344_3919 | *metE* | 4191124 | JL781 | 4191128 | 42 |
| SL1344_3922 | *udp* | 4196371 | LT2 | 4196368 | 64 |
| SL1344_3951 | *hemN* | 4232507 | LT2 | 4232507 | 61 |
| SL1344_4193 | *siiA* | 4498679 | SL1344 | 4498679 | 31 |
| SL1344_4230 | *pmrC* | 4557342 | 14028s | 4457342 | 59 |
| SL1344_4255 | *phoN* | 4584953 | 14028s | 4584953 | 24 |
| SL1344_4387 | *mgtA* | 4720007 | 14028s | 4720006 | 49 |
| SL1344_4440 | *hilE* | 4783980 | SL1344 | 4783982 | 27 |
| SL1344_4440 | *hilE* | 4783803 | SL1344 | 4783806 | 27 |

**References for Table S3:**

1. Aldridge, P., Gnerer, J., Karlinsey, J.E. and Hughes, K.T. (2006) Transcriptional and Translational Control of the *Salmonella* *fliC* Gene. *J. Bacteriol.*, **188**, 4487-4496.

2. Archer, C.D. and Elliott, T. (1995) Transcriptional control of the nuo operon which encodes the energy- conserving NADH dehydrogenase of *Salmonella typhimurium*. *J. Bacteriol.*, **177**, 2335-2342.

3. Baker, K.E., Ditullio, K.P., Neuhard, J. and Kelln, R.A. (1996) Utilization of orotate as a pyrimidine source by *Salmonella typhimurium* and *Escherichia coli* requires the dicarboxylate transport protein encoded by dctA. *J. Bacteriol.*, **178**, 7099-7105.

4. Byrne, C.R., Monroe, R.S., Ward, K.A. and Kredich, N.M. (1988) DNA sequences of the *cysK* regions of *Salmonella typhimurium* and *Escherichia coli* and linkage of the *cysK* regions to *ptsH*. *J. Bacteriol.*, **170**, 3150-3157.

5. Choi, P., Wang, L., Archer, C.D. and Elliott, T. (1996) Transcription of the glutamyl-tRNA reductase (hemA) gene in *Salmonella typhimurium* and *Escherichia coli*: role of the *hemA* P1 promoter and the *arcA* gene product. *J. Bacteriol.*, **178**, 638-646.

6. Christensen, M., Borza, T., Dandanell, G., Gilles, A.-M., Barzu, O., Kelln, R.A. and Neuhard, J. (2003) Regulation of Expression of the 2-Deoxy-D-Ribose Utilization Regulon, deoQKPX, from *Salmonella enterica* serovar *Typhimurium*. *J. Bacteriol.*, **185**, 6042-6050.

7. Craig, J.E., Boyle, D., Francis, K.P. and Gallagher, M.P. (1998) Expression of the cold-shock gene *cspB* in *Salmonella typhimurium* occurs below a threshold temperature. *Microbiology*, **144**, 697-704.

8. Darwin, K.H. and Miller, V.L. (2001) Type III secretion chaperone-dependent regulation: activation of virulence genes by SicA and InvF in *Salmonella typhimurium*. *EMBO J*, **20**, 1850-1862.

9. Elgrably-Weiss, M., Park, S., Schlosser-Silverman, E., Rosenshine, I., Imlay, J. and Altuvia, S. (2002) A *Salmonella enterica* serovar Typhimurium hemA Mutant Is Highly Susceptible to Oxidative DNA Damage. *J. Bacteriol.*, **184**, 3774-3784.

10. Espariz, M., Checa, S.K., Audero, M.E.P., Pontel, L.B. and Soncini, F.C. (2007) Dissecting the Salmonella response to copper. *Microbiology*, **153**, 2989-2997.

11. Feng, X., Walthers, D., Oropeza, R. and Kenney, L.J. (2004) The response regulator SsrB activates transcription and binds to a region overlapping OmpR binding sites at Salmonella pathogenicity island 2. *Molecular Microbiology*, **54**, 823-835.

12. Gil, F., Hernandez-Lucas, I., Polanco, R., Pacheco, N., Collao, B., Villarreal, J.M., Nardocci, G., Calva, E. and Saavedra, C.P. (2009) SoxS regulates the expression of the *Salmonella enterica* serovar Typhimurium ompW gene. *Microbiology*, **155**, 2490-2497.

13. Gillen, K.L. and Hughes, K.T. (1991) Molecular characterization of flgM, a gene encoding a negative regulator of flagellin synthesis in *Salmonella typhimurium*. *J. Bacteriol.*, **173**, 6453-6459.

14. Gunn, J.S., Alpuche-Aranda, C.M., Loomis, W.P., Belden, W.J. and Miller, S.I. (1995) Characterization of the *Salmonella typhimurium* pagC/pagD chromosomal region. *J. Bacteriol.*, **177**, 5040-5047.

15. Hamilton, S. and Miller, C.G. (1992) Cloning and nucleotide sequence of the *Salmonella typhimurium* dcp gene encoding dipeptidyl carboxypeptidase. *J. Bacteriol.*, **174**, 1626-1630.

16. Ibanez-Ruiz, M., Robbe-Saule, V., Hermant, D., Labrude, S. and Norel, F. (2000) Identification of RpoS (sigma S)-Regulated Genes in *Salmonella enterica* Serovar Typhimurium. *J. Bacteriol.*, **182**, 5749-5756.

17. Ikebe, T., Iyoda, S. and Kutsukake, K. (1999) Promoter analysis of the class 2 flagellar operons of Salmonella. *Genes & Genetic Systems*, **74**, 179-183.

18. Ikebe, T., Iyoda, S. and Kutsukake, K. (1999) Structure and expression of the fliA operon of *Salmonella typhimurium*. *Microbiology*, **145**, 1389-1396.

19. Jordan, A., Aragall, E., Gibert, I. and Barbé, J. (1996) Promoter identification and expression analysis of *Salmonella typhimurium* and *Escherichia coli* nrdEF operons encoding one of two class I ribonucleotide reductases present in both bacteria. *Molecular Microbiology*, **19**, 777-790.

20. Keane, O.M. and Dorman, C.J. (2003) The *gyr* genes of *Salmonella enterica* serovar Typhimurium are repressed by the factor for inversion stimulation, Fis. *Molecular Genetics and Genomics*, **270**, 56-65.

21. Kim, M.-j., Lim, S. and Ryu, S. (2008) Molecular Analysis of the *Salmonella typhimurium* tdc Operon Regulation. *Journal of Microbiology and Biotechnology*, **18**, 1024-1032.

22. Knodler, L.A., Celli, J., Hardt, W.-D., Vallance, B.A., Yip, C. and Finlay, B.B. (2002) Salmonella effectors within a single pathogenicity island are differentially expressed and translocated by separate type III secretion systems. *Molecular Microbiology*, **43**, 1089-1103.

23. Kutsukake, K. and Ide, N. (1995) Transcriptional analysis of the *flgK* and *fliD* operons of *Salmonella typhimurium* which encode flagellar hook-associated proteins. *Molecular and General Genetics MGG*, **247**, 275-281.

24. Lejona, S., Aguirre, A., Cabeza, M.L., Vescovi, E.G. and Soncini, F.C. (2003) Molecular Characterization of the Mg2+-Responsive PhoP-PhoQ Regulon in *Salmonella enterica*. *J. Bacteriol.*, **185**, 6287-6294.

25. Lewis, C., Skovierova, H., Rowley, G., Rezuchova, B., Homerova, D., Stevenson, A., Spencer, J., Farn, J., Kormanec, J. and Roberts, M. (2009) *Salmonella enterica* Serovar Typhimurium HtrA: regulation of expression and role of the chaperone and protease activities during infection. *Microbiology*, **155**, 873-881.

26. Lim, S., Kim, B., Choi, H.-S., Lee, Y. and Ryu, S. (2006) Fis is required for proper regulation of ssaG expression in *Salmonella enterica* serovar Typhimurium. *Microbial Pathogenesis*, **41**, 33-42.

27. Lim, S., Yun, J., Yoon, H., Park, C., Kim, B., Jeon, B., Kim, D. and Ryu, S. (2007) Mlc regulation of Salmonella pathogenicity island I gene expression via hilE repression. *Nucleic Acids Research*, **35**, 1822-1832.

28. Lim, S.Y., Yong, K.H. and Sangryeol, R. (2005) Analysis of Salmonella Pathogenicity Island 1 Expression in Response to the Changes of Osmolarity. *Journal of Microbiology and Biotechnology*, **15**, 175-182.

29. Lombardo, M.J., Lee, A.A., Knox, T.M. and Miller, C.G. (1997) Regulation of the *Salmonella typhimurium* pepT gene by cyclic AMP receptor protein (CRP) and FNR acting at a hybrid CRP-FNR site. *J. Bacteriol.*, **179**, 1909-1917.

30. Lostroh, C.P. and Lee, C.A. (2001) The HilA Box and Sequences outside It Determine the Magnitude of HilA-Dependent Activation of PprgH from Salmonella Pathogenicity Island 1. *J. Bacteriol.*, **183**, 4876-4885.

31. Main-Hester, K.L., Colpitts, K.M., Thomas, G.A., Fang, F.C. and Libby, S.J. (2008) Coordinate Regulation of Salmonella Pathogenicity Island 1 (SPI1) and SPI4 in *Salmonella enterica* Serovar Typhimurium. *Infect. Immun.*, **76**, 1024-1035.

32. McFarland, K.A. and Dorman, C.J. (2008) Autoregulated expression of the gene coding for the leucine-responsive protein, Lrp, a global regulator in *Salmonella enterica* serovar Typhimurium. *Microbiology*, **154**, 2008-2016.

33. Merighi, M., Septer, A., Carroll-Portillo, A., Bhatiya, A., Porwollik, S., McClelland, M. and Gunn, J. (2009) Genome-wide analysis of the PreA/PreB (QseB/QseC) regulon of *Salmonella enterica* serovar Typhimurium. *BMC Microbiology*, **9**, 42.

34. Miticka, H., Rowley, G., Rezuchova, B., Homerova, D., Humphreys, S., Farn, J., Roberts, M. and Kormanec, J. (2003) Transcriptional analysis of the rpoE gene encoding extracytoplasmic stress response sigma factor σE in *Salmonella enterica* serovar Typhimurium. *FEMS Microbiology Letters*, **226**, 307-314.

35. Mouslim, C. and Groisman, E.A. (2003) Control of the Salmonella ugd gene by three two-component regulatory systems. *Molecular Microbiology*, **47**, 335-344.

36. Ohnishi, K., Hasegawa, A., Matsubara, K., Date, T., Okada, T. and Kiritani, K. (1988) Cloning and nucleotide sequence of the brnQ gene, the structural gene for a membrane-associated component of the LIV-II transport system for branched-chain amino acids in *Salmonella typhimurium*. *The Japanese journal of genetics*, **63**, 343-357.

37. Olekhnovich, I.N. and Kadner, R.J. (2002) DNA-Binding Activities of the HilC and HilD Virulence Regulatory Proteins of *Salmonella enterica* Serovar Typhimurium. *J. Bacteriol.*, **184**, 4148-4160.

38. Ostrowski, J. and Kredich, N.M. (1989) Molecular characterization of the cysJIH promoters of *Salmonella typhimurium* and *Escherichia coli*: regulation by cysB protein and N-acetyl-L-serine. *J. Bacteriol.*, **171**, 130-140.

39. Ostrowski, J. and Kredich, N.M. (1991) Negative autoregulation of cysB in *Salmonella typhimurium*: in vitro interactions of CysB protein with the cysB promoter. *J. Bacteriol.*, **173**, 2212-2218.

40. Osuna, R., Lienau, D., Hughes, K.T. and Johnson, R.C. (1995) Sequence, regulation, and functions of fis in *Salmonella typhimurium*. *J. Bacteriol.*, **177**, 2021-2032.

41. Paesold, G. and Krause, M. (1999) Analysis of rpoS mRNA in Salmonella dublin: Identification of Multiple Transcripts with Growth-Phase-Dependent Variation in Transcript Stability. *J. Bacteriol.*, **181**, 1264-1268.

42. Plamann, L.S. and Stauffer, G.V. (1987) Nucleotide sequence of the *Salmonella typhimurium* metR gene and the metR-metE control region. *J. Bacteriol.*, **169**, 3932-3937.

43. Rajkumari, K., Ishihama, A. and Gowrishankar, J. (1997) Evidence for transcription attenuation rendering cryptic a sigmaS- dependent promoter of the osmotically regulated proU operon of *Salmonella typhimurium*. *J. Bacteriol.*, **179**, 7169-7173.

44. Richter-Dahlfors, A.A. and Andersson, D.I. (1991) Analysis of an anaerobically induced promoter for the cobalamin biosynthetic genes in *Salmonella typhimurium*. *Molecular Microbiology*, **5**, 1337-1345.

45. Schechter, L.M., Damrauer, S.M. and Lee, C.A. (1999) Two AraC/XylS family members can independently counteract the effect of repressing sequences upstream of the hilA promoter. *Molecular Microbiology*, **32**, 629-642.

46. Schechter, L.M., Jain, S., Akbar, S. and Lee, C.A. (2003) The Small Nucleoid-Binding Proteins H-NS, HU, and Fis Affect hilA Expression in *Salmonella enterica* Serovar Typhimurium. *Infect. Immun.*, **71**, 5432-5435.

47. Sharma, C.M., Darfeuille, F., Plantinga, T.H. and Vogel, J.r. (2007) A small RNA regulates multiple ABC transporter mRNAs by targeting C/A-rich elements inside and upstream of ribosome-binding sites. *Genes & Development*, **21**, 2804-2817.

48. Shi, Y., Latifi, T., Cromie, M.J. and Groisman, E.A. (2004) Transcriptional Control of the Antimicrobial Peptide Resistance ugtL Gene by the Salmonella PhoP and SlyA Regulatory Proteins. *Journal of Biological Chemistry*, **279**, 38618-38625.

49. Soncini, F.C., Vescovi, E.G. and Groisman, E.A. (1995) Transcriptional autoregulation of the *Salmonella typhimurium* phoPQ operon. *J. Bacteriol.*, **177**, 4364-4371.

50. Sorensen, K.I., Baker, K.E., Kelln, R.A. and Neuhard, J. (1993) Nucleotide pool-sensitive selection of the transcriptional start site in vivo at the *Salmonella typhimurium* pyrC and pyrD promoters. *J. Bacteriol.*, **175**, 4137-4144.

51. Stapleton, M.R., Norte, V.A., Read, R.C. and Green, J. (2002) Interaction of the *Salmonella typhimurium*Transcription and Virulence Factor SlyA with Target DNA and Identification of Members of the SlyA Regulon. *Journal of Biological Chemistry*, **277**, 17630-17637.

52. Sulavik, M.C., Dazer, M. and Miller, P.F. (1997) The *Salmonella typhimurium* mar locus: molecular and genetic analyses and assessment of its role in virulence. *J. Bacteriol.*, **179**, 1857-1866.

53. Tartaglia, L.A., Storz, G. and Ames, B.N. (1989) Identification and molecular analysis of oxyR-regulated promoters important for the bacterial adaptation to oxidative stress *Journal of Molecular Biology* **210**, 709-719.

54. Theisen, M., Kelln, R.A. and Neuhard, J. (1987) Cloning and characterization of the pyrF operon of *Salmonella typhimurium*. *European Journal of Biochemistry*, **164**, 613-619.

55. Tinker, J.K., Hancox, L.S. and Clegg, S. (2001) FimW Is a Negative Regulator Affecting Type 1 Fimbrial Expression in *Salmonella enterica* Serovar Typhimurium. *J. Bacteriol.*, **183**, 435-442.

56. Waldminghaus, T., Heidrich, N., Brantl, S. and Narberhaus, F. (2007) FourU: a novel type of RNA thermometer in Salmonella. *Molecular Microbiology*, **65**, 413-424.

57. Walthers, D., Carroll, R.K., Navarre, W.W., Libby, S.J., Fang, F.C. and Kenney, L.J. (2007) The response regulator SsrB activates expression of diverse Salmonella pathogenicity island 2 promoters and counters silencing by the nucleoid-associated protein H-NS. *Molecular Microbiology*, **65**, 477-493.

58. Wong, D.K.H., Morris, C., Lam, T.L., Wong, W.K.R. and Hackett, J. (1999) Identification of O-antigen polymerase transcription and translation start signals and visualization of the protein in *Salmonella enterica* serovar Typhimurium. *Microbiology*, **145**, 2443-2451.

59. Wosten, M.M.S.M. and Groisman, E.A. (1999) Molecular Characterization of the PmrA Regulon. *Journal of Biological Chemistry*, **274**, 27185-27190.

60. Xu, K. and Elliott, T. (1993) An oxygen-dependent coproporphyrinogen oxidase encoded by the hemF gene of *Salmonella typhimurium*. *J. Bacteriol.*, **175**, 4990-4999.

61. Xu, K. and Elliott, T. (1994) Cloning, DNA sequence, and complementation analysis of the *Salmonella typhimurium* hemN gene encoding a putative oxygen-independent coproporphyrinogen III oxidase. *J. Bacteriol.*, **176**, 3196-3203.

62. Yanagihara, S., Iyoda, S., Ohnishi, K., Iino, T. and Kutsukake, K. (1999) Structure and transcriptional control of the flagellar master operon of *Salmonella typhimurium*. *Genes & Genetic Systems*, **74**, 105-111.

63. Zaharik, M.L., Lamb, S.S., Baker, K.E., Krogan, N.J., Neuhard, J. and Kelln, R.A. (2007) Mutations in *yhiT* enable utilization of exogenous pyrimidine intermediates in *Salmonella enterica* serovar Typhimurium. *Microbiology*, **153**, 2472-2482.

64. Zolotukhina, M., Ovcharova, I., Eremina, S., Errais Lopes, L. and Mironov, A.S. (2003) Comparison of the structure and regulation of the *udp* gene of *Vibrio cholerae, Yersinia pseudotuberculosis*, *Salmonella typhimurium*, and *Escherichia coli*. *Research in Microbiology*, **154**, 510-520.

65. Pfeiffer V, Sittka A, Tomer R, Tedin K, Brinkmann V, **Vogel J** (2007) A small
 noncoding RNA of the invasion gene island (SPI-1) represses outer membrane
 protein synthesis from the Salmonella core genome. ***Molecular Microbiology,***
 **66(5):**1174-1191

**Table S4.** **Probes and primers used for detection of ncRNAs.**

| **Small RNA** | **Probe sequence (5’ – 3’)** |
| --- | --- |
| InvR | CGTAAGAGACAAATGGCCAACCTA |
| GcvB | GCCCATCAGAACACGCATTCC |
| RprA | CACTCAGGGGATTTCCATGCTTATAAA |
| STnc1020 | CTTGATTACCACAACCACATCACGAAG |
| sRNA1 (SLnc0011) | GGTCACCAACGGCTTTCTGGTG |
| sRNA2 (SLaRNA0183) | CGAATGAGGGGCTAACTTGCAG |
| sRNA3 (SLnc0027) | GGAAATACCGGCTCTAACGGATAC |
| 5S | CCACACTACCATCGGCGCTAC |
| sRNA9 (SLaRNA0361) | CCTGGTGCATCATAACCCTTTCC |
| SLP1a (SLP1_ncRNA3) | CATGAAGGAGTATCAATAATGTC |
| SLP1b (SLP1_ncRNA6) | CATCACAAATCACCAACCGG |
| SLP2a (SLP2_ncRNA1) | CGGAACGACGAAAGATAGTGG |
| SLP2b (SLP2_ncRNA12) | CTATCTATGGAGATTATGCCA |

| **Antisense RNA** | **Primer sequence (5’ – 3’)** |
| --- | --- |
| SLaRNA0330_PCR | ATATCTAACAGCTGCTTTTCC |
| SLARNA0200_PCR | ATTACTCATCACTATCTTCC |
| SLaRNA0398_PCR | ATTCGTCCAGGGGCGTGTG |
| SLaRNA0293_PCR | CTACGGTGTGCCTGCGTTGC |
| SLaRNA0255_PCR | CATGCGATGGTGAGTACAAA |
| SLaRNA0288_PCR | GCGCAGCGCCGTCAGGCATTA |

Probe sequences used for Northern blot detection of sRNAs and the sequences for PCR primers used for detection of asRNAs.
