# Supplementary material for: The architecture and ppGpp-dependent expression of the primary transcriptome of Salmonella Typhimurium during invasion gene expression
Source: BMC Genomics. 2012 Jan 17;13:25. doi: 10.1186/1471-2164-13-25 (PMC3293720; doi:10.1186/1471-2164-13-25)
Supplement: Additional file 3 — Supplementary Figures S1, S2, S3, S4, S5, S6, S7, S8, S9. Figure S1: 5' RACE identification of transcriptional start sites. Figure S2: Functional category analysis of 1932 promoters of annotated SL1344 ORFs that contain a predicted -10 motif. Figure S3: Functional category analysis of 1932 promoters of annotated SL1344 ORFs that contain a predicted -10 and -35 motif. Figure S4: Functional category analysis of 264 promoters of annotated SL1344 ORFs that contain conserved motif 1. Figure S5: Northern Blot detection of non-coding RNAs. Figure S6: Adapter assisted PCR detection of asRNAs. Figure S7: Functional category analysis of ORFs opposite to candidate asRNAs. Figure S8: Growth curves for S. Typhimurium SL1344 wild-type ΔrelAΔspoT strains. Figure S9: Invasion of S. Typhimurium SL1344 wild-type and isogenic ΔrelAΔspoT strains in HeLa cells at 2 h and intracellular replication at 6 h post-infection. [file 1471-2164-13-25-S3.DOC]

**
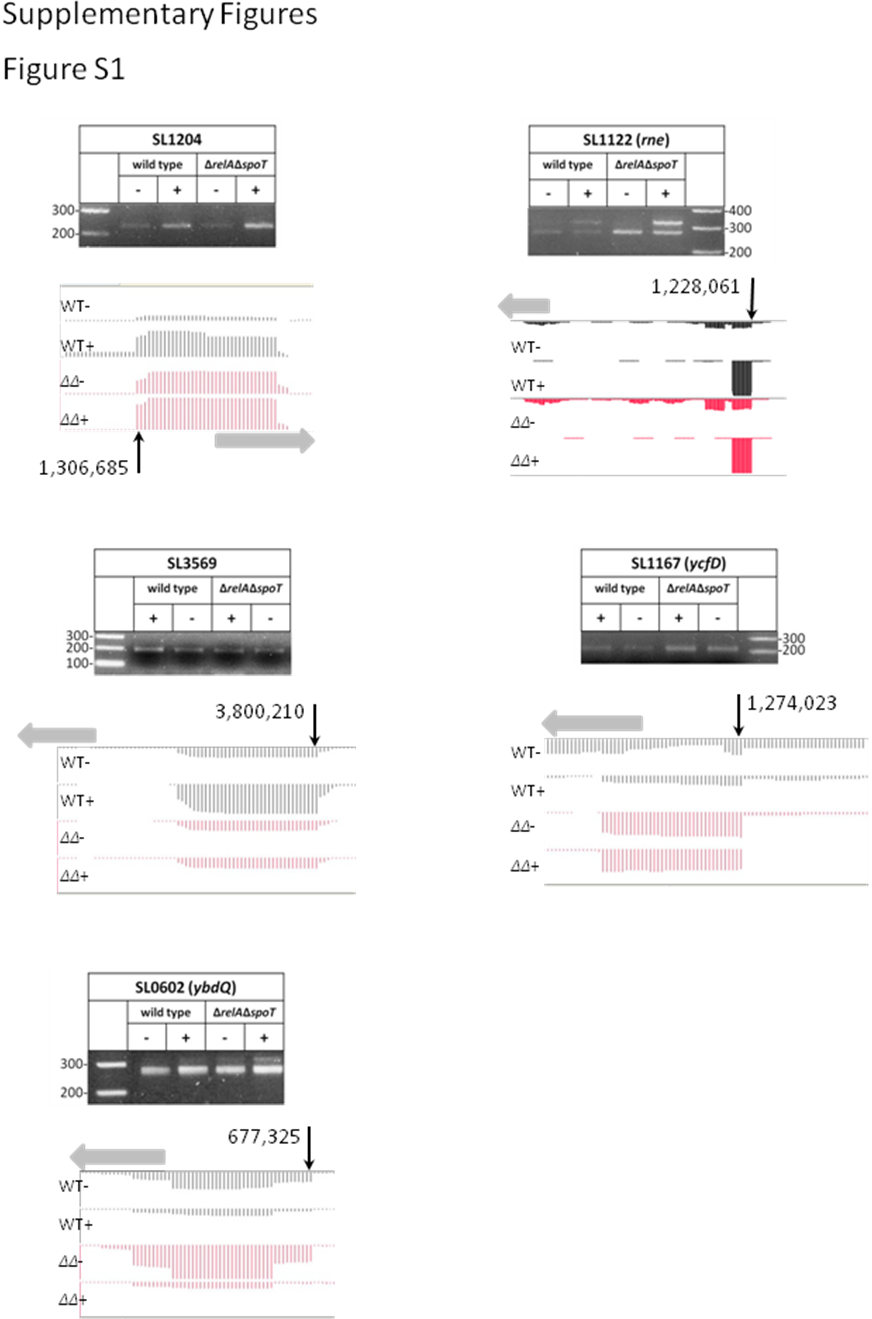
**

**A**

**B**

**C**

**D**

**E**

**5’ RACE identification of transcriptional start sites.** 5’ RACE was used to confirm TSSs for the following SL1344 genes: **(A)** SL1204, **(B)** SL1122, **(C)** SL3569, **(D)** SL1167, **(E)** SL0602. PCR products obtained from 5’ RACE were separated by agarose gel electrophoresis. The gel images are shown above screen shots of the Integrated Genome Browser (IGB) which shows the corresponding dRNA-seq identified TSSs. The IGB screen shots, showing the relative levels of non-enriched and enriched reads were used to identify the positions of the TSSs. Above the gel image, plus or minus symbols indicate Tobacco Acid Pyrophosphatase (TAP) treatment or mock control respectively. The arrows indicate the locations of the TSSs which were confirmed by sequencing of 5’ RACE products. Grey horizontal arrows in the IGB screen shots show a portion of the gene for which TSSs have been identified. In the IGB screen shots, a plus symbol indicates RNA samples enriched for primary TSSs and a minus symbol indicates non-enriched samples; wt and ∆∆ refer to *S*. Typhimurium SL1344 wild-type and ∆*relA*∆*spoT* strains respectively. In several of the RNA-seq identified TSSs we noted that read numbers were the same or higher in the non-enriched compared to enriched samples, however the position of the TSSs were the same. For two of these TSSs (SL1169 and SL0602) we used 5’ RACE to confirm that the TSSs were located at the positions identified from the dRNA-seq inspection. It is unclear why the read numbers within these promoters are the same (SL1167) or higher (SL0602) in the non-enriched compared to the enriched samples, however we suggest this may be a consequence of differential processing. Of the two bands observed for SL1122 within the ∆*relA*∆*spoT* strain **(B)**, the higher molecular weight band (marked with a black arrow) corresponds to the primary TSS whereas we suggest the smaller molecular weight band may be a stable, processed fragment.

**Figure S2.**

**
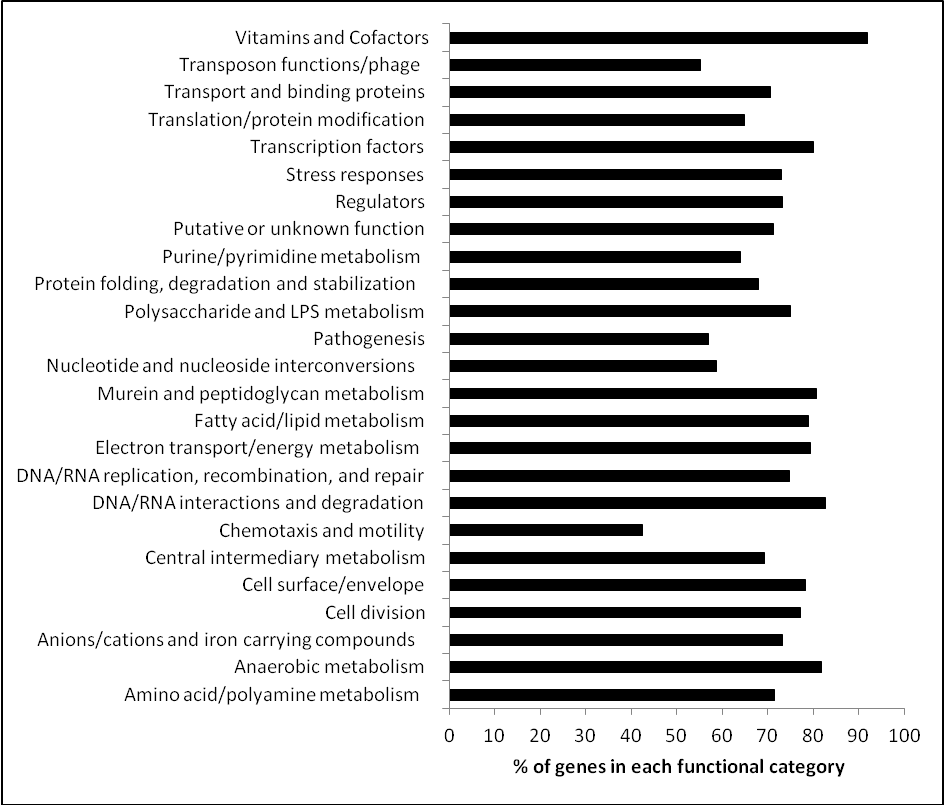
**

**Functional category analysis of 1932 promoters of annotated SL1344 ORFs that contain a predicted -10 motif.** Functional categories were assembled from the Kyoto Encyclopedia of Genes and Genomes (KEGG; www.genome.jp/kegg) and The Comprehensive Microbial Resource (CMR) at the J. Craig Ventner Institute (http://cmr.jcvi.org/tigr-scripts/CMR/CmrHomePage.cgi) and a manual inspection based on the published literature.

**Figure S3**

**
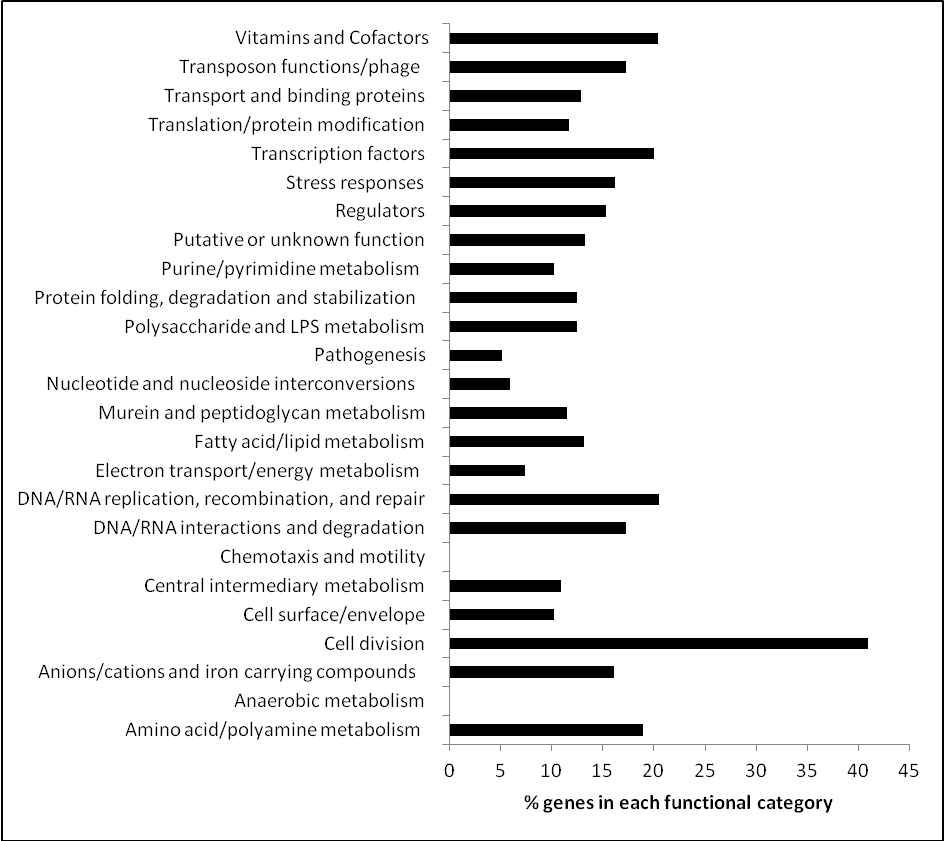
**

**Functional category analysis of 1932 promoters of annotated chromosomal SL1344 ORFs that contain a predicted -10 and -35 motifs.**

**Figure S4**

**
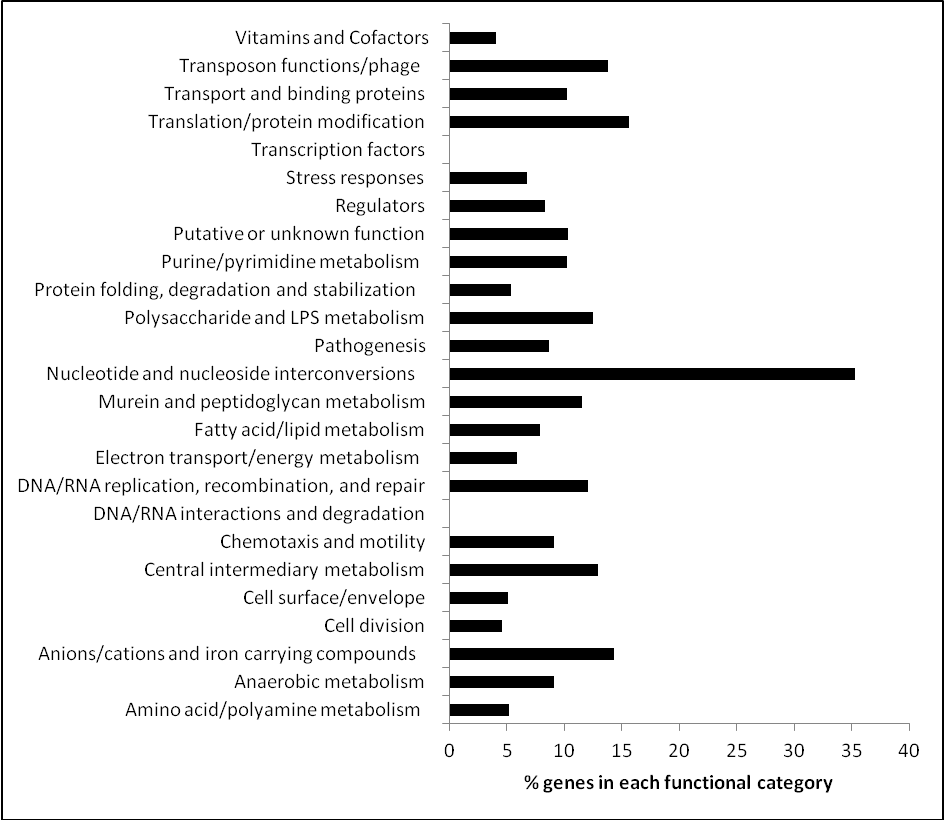
**

**Functional category analysis of 264 promoters of annotated SL1344 ORFs that contain conserved motif 1.**

**Figure S5.**


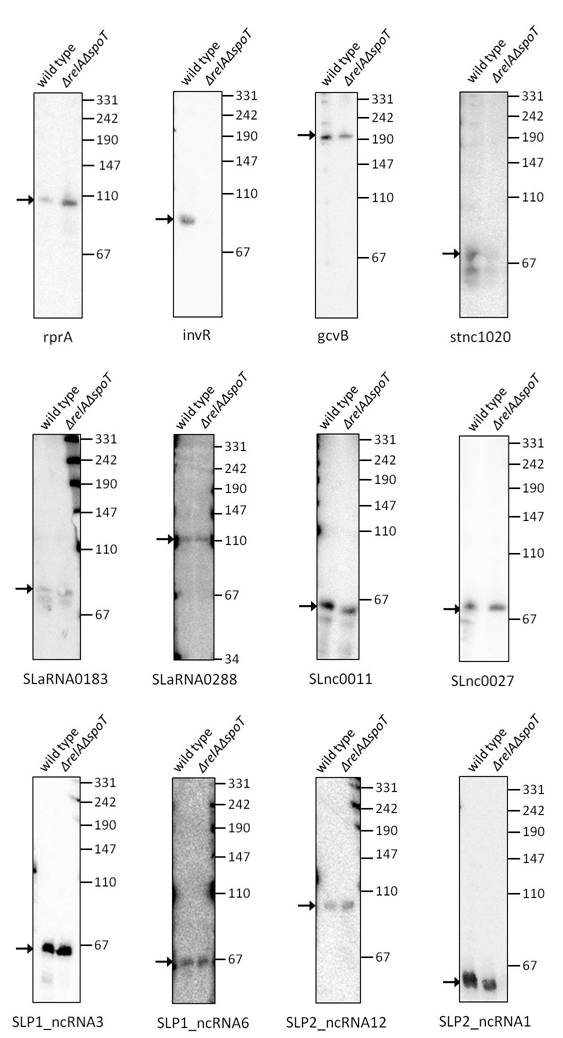


**C**

**A**

**B**

**Northern Blot detection of non-coding RNAs. (A)** Known and predicted sRNAs (RprA, InvR, GcvB, STnc1020), **(B)** new antisense (SLasRNA0183) and sRNAs (SLncRNA0288, SLnc0011, SLnc0027) **(C)** Plasmid encoded sRNAs (SLP1_ncRNA3, SLP1_ncRNA6, SLP2_ncRNA12, SLP2_ncRNA1). Northern Blotting was carried out as described in Materials and Methods. Blots were stripped and re-probed with 5S RNA probe to confirm loading of equal quantities of wild type and Δ*relA*Δ*spoT* RNA (results not shown). Arrows indicate positions of bands which correspond to the approximate expected transcript lengths derived from dRNA-seq data. The double bands observed for samples stnc1020, SLasRNA0183 and SLnc0011 may be due to processing or degradation.

**Figure S6.**


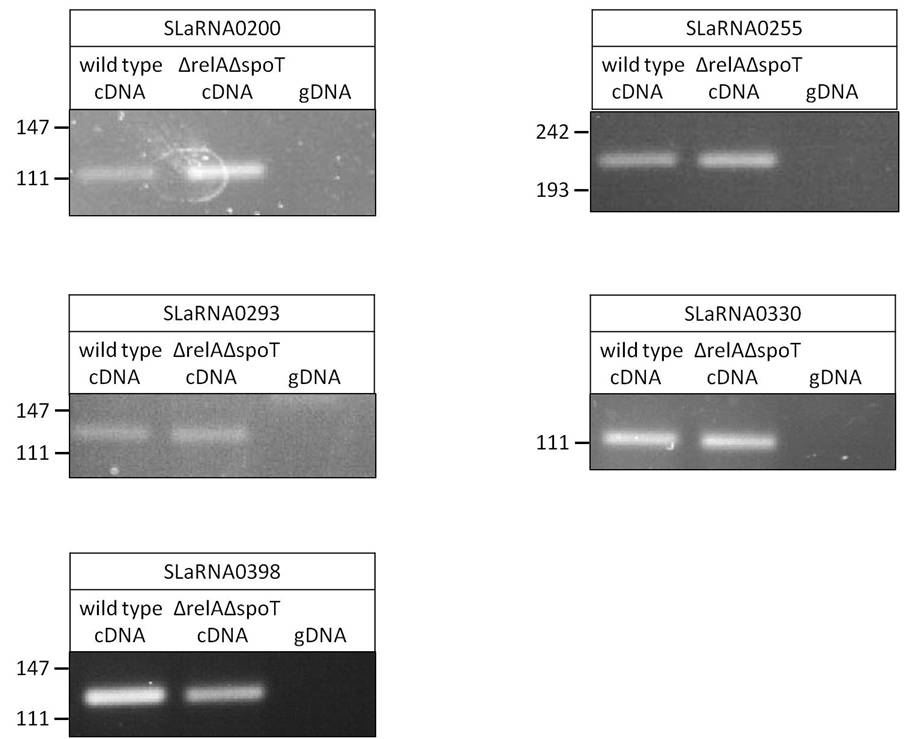


**Adapter assisted PCR detection of asRNAs.** The transcripts corresponding to 5 asRNAs identified from a manual inspection dRNA-seq data were confirmed to be present within independently isolated RNA samples using adaptor assisted RT-PCR (Materials and Methods).

**Figure S7.**

**
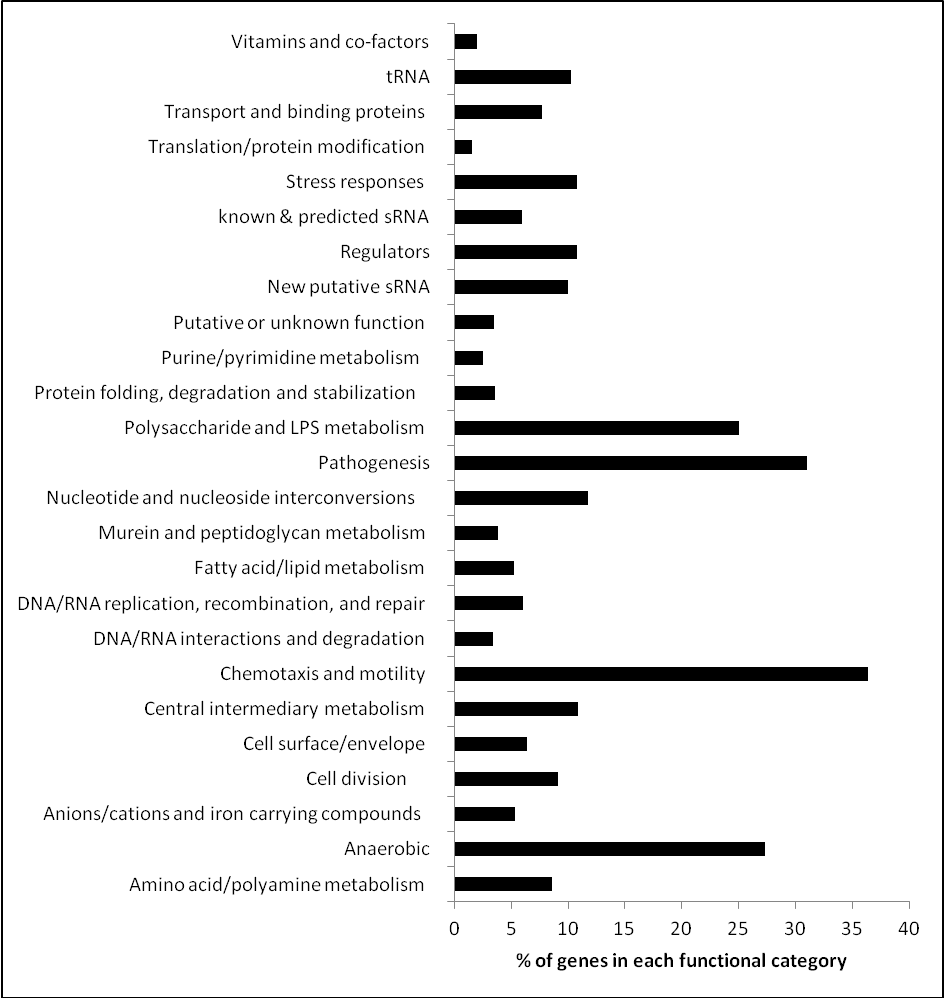
**

**Functional category analysis of ORFs opposite to 302 candidate asRNAs**

**Figure S8.**

**
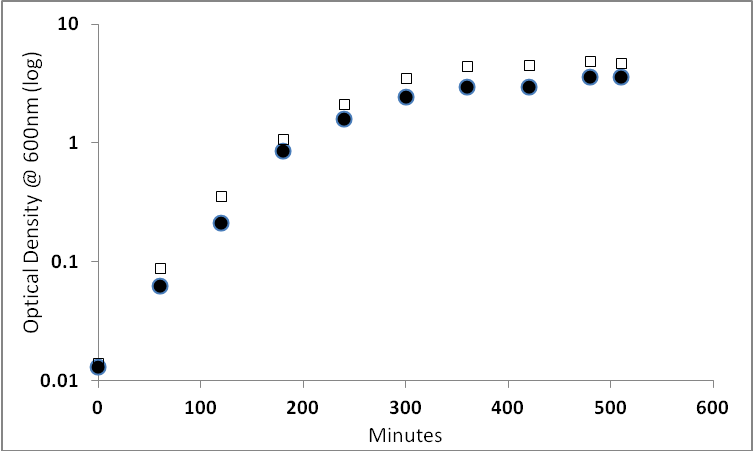
**

**Growth curves for *S*. Typhimurium SL1344 wild-type (closed circles) Δ*relA*Δ*spoT* strains (open squares).** Typical growth curves were performed on aerobically grown LB cultures at 37^0^C in a water bath shaking at 250 rpm. Under these conditions growth of wild-type and ∆*rel*A∆*spoT* strains were almost identical, however the ∆*rel*A∆*spoT* strain attained a slightly higher final density, in accordance with the findings of Pizarro-Cerdá and Tedin, 2004. *Molecular Microbiology,* **52**: 1827–1844.

**Figure S9.**

**
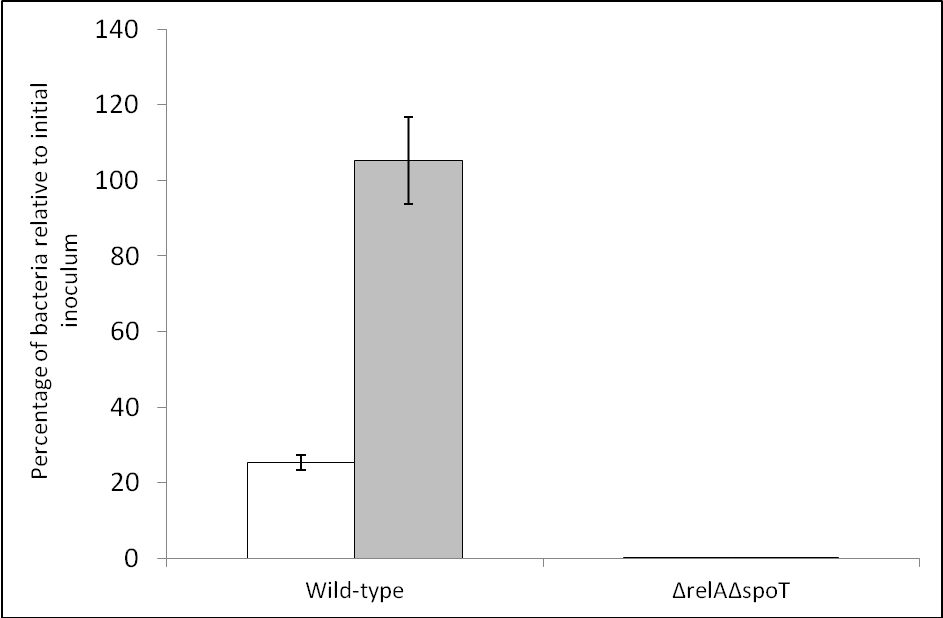
**

**Invasion of *S*. Typhimurium SL1344 wild-type and isogenic Δ*relA*Δ*spoT* strains in HeLa cells at 2h and intracellular replication at 6h post-infection.** Infection protocols were carried out according to Hautefort *et al* 2008. *Cell. Microbiol*. **10:** 958-984. The bacteria were grown aerobically at 37^0^C in LB to an OD_600_ of 2.1 at 250rpm in a shaking water bath. Previous work has shown that *S*. Typhimurium grown to early stationary phase induces SPI1 expression (Song *et al.,* 2010. *J. Microbiol* **48**: 89-95). The open and shaded columns represent percentage of intracellular bacteria after 2 hours (invasion) and 6 hours post-infection (intracellular replication) compared to the initial inoculum. Data are from three independent biological replicate experiments**.**
